# Measuring pathway database coverage of the phosphoproteome

**DOI:** 10.7717/peerj.11298

**Published:** 2021-05-25

**Authors:** Hannah Huckstep, Liam G. Fearnley, Melissa J. Davis

**Affiliations:** 1 Division of Bioinformatics, The Walter and Eliza Hall Institute of Medical Research, Parkville, Victoria, Australia; 2 Department of Medical Biology, Faculty of Medicine, Dentistry and Health Sciences, University of Melbourne, Parkville, Victoria, Australia; 3 Division of Population Health, Walter and Eliza Hall Institute of Medical Research, Parkville, Victoria, Australia; 4 Department of Clinical Pathology, Faculty of Medicine, Dentistry and Health Sciences, University of Melbourne, Melbourne, Victoria, Australia

**Keywords:** Phosphoproteomics, Databases, Proteomics, Bioinformatics

## Abstract

Protein phosphorylation is one of the best known post-translational mechanisms playing a key role in the regulation of cellular processes. Over 100,000 distinct phosphorylation sites have been discovered through constant improvement of mass spectrometry based phosphoproteomics in the last decade. However, data saturation is occurring and the bottleneck of assigning biologically relevant functionality to phosphosites needs to be addressed. There has been finite success in using data-driven approaches to reveal phosphosite functionality due to a range of limitations. The alternate, more suitable approach is making use of prior knowledge from literature-derived databases. Here, we analysed seven widely used databases to shed light on their suitability to provide functional insights into phosphoproteomics data. We first determined the global coverage of each database at both the protein and phosphosite level. We also determined how consistent each database was in its phosphorylation annotations compared to a global standard. Finally, we looked in detail at the coverage of each database over six experimental datasets. Our analysis highlights the relative strengths and weaknesses of each database, providing a guide in how each can be best used to identify biological mechanisms in phosphoproteomic data.

## Introduction

Phosphorylation is a reversible post-translational modification (PTM) capable of controlling numerous aspects of a protein’s function. The effects of phosphorylation are widespread and central to cellular signal transduction, with aberrant protein phosphorylation known to be both a cause and result of many diseases such as cancer and diabetes (Cohen, 2001). A network of kinases and phosphatases finely regulate protein phosphorylation and in turn, cellular signalling. These enzymes offer a great opportunity for targeted therapies to treat disease. Currently, more than 28 kinase inhibitors have been approved for clinical use and up to one-third of protein targets being developed in the pharmaceutical industry are directly related to kinases (Rask-Andersen et al., 2014; Bhullar et al., 2018). It has been found over years of targeted cancer therapy that the clinical success rate of kinase inhibitors exceeded other cancer therapies demonstrating higher selectivity and lower cytotoxicity (Walker & Newell, 2009). These achievements highlight the importance of understanding phosphorylation networks in disease and in normal function.

Continuous advances in mass spectrometry have enabled large-scale identification and profiling of phosphosites across cell lines, tissues and species. The number of protein phosphosites is estimated at 230,000 in humans and 156,000 in mice (Vlastaridis et al., 2017). Yet these discoveries are vastly outpacing our understanding of the function and regulation of these PTMs. Phosphorylating kinases are known for only 5% of phosphosites, and a negligible number of phosphosites have recorded the implications of the phosphorylation event (Needham et al., 2019). Disagreement exists over the proportion of phosphosites predicted to be functionally important based on being evolutionarily conserved. Studies report anywhere from 35% (Landry, Levy & Michnick, 2009) to 65% (Gray & Kumar, 2011) of phosphosites are conserved, highlighting the difficulty in estimating the functional proportion of all known phosphosites. Despite this discrepancy, these reports agree that the proportion of functional phosphosites is much higher than the 5% that have been annotated to date.

In principle, the abundance of detailed phosphoproteomics data naturally lends itself to data-driven modelling approaches such as principal component analysis (PCA) or clustering (Janes & Yaffe, 2006). Drawing functional conclusions from these methods has not been as simple as anticipated. Many limitations of varying complexity stand in the way of elucidating phosphosite specific mechanisms. First, a protein phosphorylation can have a potential activating or inhibiting effect, which cannot be easily captured through typical high-throughput data alone. Next, operating under the conservative assumption that approximately 65% of phosphosites are not conserved and therefore have no functional consequence, there is an increased difficulty in distinguishing which few remaining phosphosites are functionally relevant (Lienhard, 2008; Levy, Michnick & Landry, 2012). Finally, it was found that 75% of significantly phosphorylated proteins have at least two phosphosites that are oppositely modulated (Olsen et al., 2006) making the use of high-throughput data alone largely unsuitable to determine which phosphosite modulates which function. On the other hand, network-driven methods which make use of previously established literature-derived knowledge of signalling, offer a more straightforward approach (Sacco, Perfetto & Cesareni, 2018).

Many databases capable of analysing phosphoproteomics broadly fit into three categories; Pathway databases, Protein–Protein Interaction (PPI) databases, and Phosphoproteomics focused databases. These databases were created for differing purposes and are typically chosen for differing types of analysis. However, each category holds signalling information pertaining to how a phosphorylation may affect signalling cascades. Each category has broad strengths and weaknesses in their capacity to analyse phosphoproteomics data as well as variable information and conclusions that can be drawn from an analysis. For example, a phosphoproteomics database would be best-suited to investigating upstream kinases (either experimentally derived or predicted) of a phosphosite of interest, while a pathway database can be used to investigate the phosphosite’s potential functional role in a particular phenotype or cell of interest. A PPI database captures information on potential interactions that might suggest complexes that phosphoprotein may be involved in, or potential interactions outside of a pathway context. These databases, however, can overlap as some PPI and pathway databases hold kinase-substrate (K-S) information, some pathway databases hold comprehensive complex information, and some phosphorylation databases hold disease and pathway annotations. These databases can be used both individually and in conjunction to provide a comprehensive analysis capable of developing hypotheses around individual phosphorylation signalling mechanisms.

Literature-derived, pathway-oriented databases such as the Kyoto Encyclopedia of Genes and Genomics (KEGG) (Kanehisa & Goto, 2000), Reactome (Joshi-Tope et al., 2005), WikiPathways (Slenter et al., 2018), and the SIGnaling Network Open Resource (SIGNOR) (Perfetto et al., 2016) have proven to be immensely useful in providing functional insight for other types of ‘omic data. This has been largely driven through the development of enrichment methods using pathway-level information (Khatri, Sirota & Butte, 2012). Many tools have been developed to perform such analyses (Yu et al., 2012; Young et al., 2010; Subramanian et al., 2005; Wu et al., 2010). Another popular method for analysis and interpretation of ‘omics data is to overlay or map data onto pathways. This technique has been successful in analysing data from metabolomics (Chagoyen & Pazos, 2013), genomics (Ramanan et al., 2012), transcriptomics (Wang et al., 2019) and also has been successful in numerous examples with phosphoproteomics data (Satpathy et al., 2015; Humphrey et al., 2013; Rudolph et al., 2016). These database resources are dependent upon curation and are constantly updating molecular interactions making them an invaluable source of current literature-derived knowledge. However, they are not without their own limitations as the curation process tends to result in an overrepresentation of well-studied proteins and a lack of lesser-known proteins, in particular phosphoproteins. In addition, in a comparison of phosphosite databases Sacco et al. found that many phosphosites that had been identified and characterized by low-throughput experiments (approximately 9% of PhosphoSitePlus at the time) could not be found in a typical phosphoproteomics experiment. They hypothesized that this may be due to a number of reasons including abundance, large sample complexity and the stochastic nature of peptide selection (Sacco, Perfetto & Cesareni, 2018).

A recent study comparing PPI databases found 375 resources in their literature search and observed that PubMed timeline data indicated a steady increase in research articles on PPIs across the years (Bajpai et al., 2020). Resources such as the Human Protein Reference Database (HPRD) (Peri et al., 2004; Keshava Prasad et al., 2009), the Biological General Repository for Interaction Datasets (BioGRID) (Stark et al., 2006), and the Search Tool for Retrieval of Interacting Genes/Proteins (STRING) (Szklarczyk et al., 2019) are incredibly useful resources that enable the analysis of interaction data in various contexts. PPI databases tend to be quite large as a result of their derivation from high-throughput experimental data. This may be an advantage when looking for novel associations highlighted when measurements are mapped onto the data. As with pathway databases, PPIs are not without their limitations, as they typically represent an oversimplified depiction of cellular signalling making interpretation of the functional consequences of molecular perturbations difficult.

Popular phosphorylation focused databases such as PhosphoSitePlus (Hornbeck et al., 2015), PHOSIDA (Gnad et al., 2007), Phospho.ELM (Diella et al., 2004) and qPhos (Yu et al., 2019) host many phosphosites and act as repositories for both low and high-throughput data. Some have tools for motif analysis and phosphosite prediction but rarely contain information on the function of a phosphosite. qPhos is amongst the largest such databases and contains quantitative information for almost 200,000 non-redundant phosphorylation sites as well as the cell-type and temporal information. An extensive review of phosphoproteomics resources can be found here (Savage & Zhang, 2020). Although these databases can be extremely useful in their own right, the lack of functional annotations makes their use in elucidating phosphosite signalling mechanisms rather limited. Of those databases listed above, the exception is PhosphoSitePlus, which also contains a network of experimentally observed kinase-substrate relationships. Moving away from databases acting as phosphosite repositories, many databases holding signalling information in the form of a K-S networks exist such as RegPhos (Huang et al., 2014), PhosphoNet (Safaei et al., 2011) and Phosphonetworks (Hu et al., 2014). Though they are limited to K-S interactions, they have been proven useful in providing phosphoproteomics mechanistic insight either on their own or while integrated into other databases (Rohrs et al., 2018; McGuire et al., 2017; Tong et al., 2019). One method developed by Sacco et al. (2016b) starts by overlaying their data onto a literature-derived, pathway-oriented database, followed by filtering the network to an interpretable size based on a set of rules they developed. This method was successfully implemented in a phosphoproteomics context by mapping their data onto the K-S network from PhosphoSitePlus to discover novel mechanistic insights into phosphorylation mediated insulin signalling (Sacco et al., 2016a).

This work aims to systematically investigate the current landscape of resources suitable for the network-based analysis of phosphoproteomic data (as defined by our selection criteria, see Methods and Table S1). Specifically, we are interested in how well equipped each database is to uncover mechanisms of phosphorylation and connect phosphorylation events to a signalling network or pathway. The pathway databases we compare here are Reactome, KEGG, WikiPathways and SIGNOR. The PPI databases we compare are BioGRID and HPRD. PhosphoSitePlus was also included as it contains the largest subset of phosphoproteomic signalling-related proteins. We first analysed the proteome coverage of each database followed by the phosphorylation coverage of a subset of the above databases. Next, we explored the consistency between the database’s phosphorylation annotations and the amino acid residue found in UniProt’s (Bateman et al., 2017) protein sequence. Finally, we assessed the capability of each database in mapping experimental phosphoproteomics datasets of varying backgrounds.

## Materials and Methods

### Systematic literature review of public databases

There is an abundance of pathway databases currently available for public use in interpreting biological data (Kanehisa & Goto, 2000; Joshi-Tope et al., 2005; Slenter et al., 2018; Zhou et al., 2012; Wishart et al., 2006; Pratt et al., 2015; Szklarczyk et al., 2019; Cowley et al., 2012). However, they tend to differ from each other in several properties. To address this, we systematically compared 29 databases and produced a selection criterion for inclusion in our analysis (Table S1, Supporting Information).

To be included in this comparison, each database had to fit the following criteria:

- Have phosphorylation information
- Have a connected signalling network component
- Be updated yearly, and if not must have been widely used and be widely cited (1,000 minimum)
- Freely accessible
- Downloadable

The pathway databases which met these criteria were HPRD, BioGRID, SIGNOR, Reactome, KEGG, PhosphoSitePlus and WikiPathways.

### Data retrieval and processing

Since many of these databases were represented in differing formats and some with unique identifiers, several processing steps were conducted as needed per database to extract protein UniProt accessions and phosphosites as outlined in Fig. S1, Supporting Information. Dates of database access are outlined in Table S2, Supporting Information. For those databases available in a BioPax format, they were first converted into a Neo4j graph database object (Neo4j, 2020) (Schema described in Fig. S2, Supporting Information). The Neo4j graph database implementations were built and used to allow for quick and easy extraction of relevant information as described in more detail below.

### Protein

For IMEX, qPhos and, UniProt and both Gene Ontology (GO) categories the data was available in TSV format with UniProt accessions easily extractable. Each database downloaded in the BioPax format (Reactome and PhosphoSitePlus) was converted into a Neo4j graph database in Java, where the list of UniProt accessions were extracted. SIGNOR’s API was used to download the list of UniProt IDs involved in the signalling network. BioGRID’s PPI file was downloaded in TSV format directly from the website with RefSeq IDs from the signalling portion of the database being taken. HPRD legacy files were downloaded from the Integrated Network and Dynamical Reasoning Assembler (INDRA) python package documentation (Gyori et al., 2017). For HPRD, RefSeq identifiers were taken, once again only from the signalling portion of the database. We consider the interaction network portion of HPRD and BioGRID, as a source of connected proteins with potential signalling function. For both KEGG and WikiPathways only the identifiers belonging to a pathway were downloaded as we were only interested in proteins involved in signalling. The KEGG database contained only KEGG identifiers, and the WikiPathways download contained only Entrez IDs. All sets of identifiers were converted to UniProt accessions. No identifier type mapped cleanly to UniProt IDs, so in cases where more than one UniProt accession was found, only the reviewed UniProt accession was taken. If there were multiple reviewed UniProt accessions found, the UniProt accession with the longest protein sequence was selected. If no reviewed UniProt accession was found, the unreviewed UniProt accession with the longest sequence was taken. This was done to maintain a one-to-one ratio of alternate identifiers to UniProt accessions. Because some resources annotate pathways with gene level identifiers, we remove annotated isoforms from other resources in order to maintain a fair comparison.

Once the list of UniProt accessions were obtained for each resource, each was updated to the latest version of UniProt (Jan. 2021). In this instance, ‘update’ refers to converting all secondary accessions to primary accessions, removing all non-human identifiers and removing identifiers that are classified as obsolete in UniProt.

### Phosphosites

In Reactome and PhosphoSitePlus, the phosphosites were extracted from the neo4j graph databases generated for extracting the UniProt accessions mentioned above. For qPhos, the set of all phosphosites were extracted from the main data file downloaded. Phosphosites from HPRD were extracted from the separate PTM file and connected to UniProt IDs converted from RefSeq IDs with only phosphosites belonging to the signalling portion of the database taken. BioGRID’s phosphosites were also extracted from a separate PTM file and connected to UniProt IDs converted from RefSeq IDs only from the signalling portion of the database. For SIGNOR, the human PTM file was downloaded directly from the website and processed. Phosphosites from UniProt were extracted directly from the January 2021 downloaded TSV file. Only phosphosites from reviewed UniProt accessions were used in this study. Upon further investigation, phosphorylations listed in KEGG had no specific site annotations. WikiPathways contained multiple phosphosite annotations in multiple formats, making it impossible to extract them all. As with above, since each phosphosite was associated with a single UniProt accession, the UniProt accessions were updated to reflect only human and current UniProt accessions and isoforms were removed.

### Consistency analysis

To perform the consistency analysis the neo4j graph database version of Reactome and PhosphoSitePlus were used. For SIGNOR, HPRD and BioGRID the list of unique phosphosites retrieved from each respective database file was used. In each database, all PTM’s belonging to a unique UniProt id were gathered into a set. A phosphosite in this manuscript is defined as a single amino acid reported to be phosphorylated and its position as reported in the database. We do not take into surrounding sequence or attempt to standardise or harmonize this information across databases beyond what is described in the methods. Then, for each UniProt ID the canonical sequence and all isoform sequences were retrieved from UniProt. Next, for each PTM annotation, if the amino acid at the annotated position was the same in the canonical UniProt sequence it was called a match; for example, if Reactome contains an annotation on Protein X that indicates a phosphorylation on a threonine at location 128 and UniProt has a threonine at position 128 in its sequence this is a match. If, however, the annotated residue and the canonical residue didn’t match but another phosphorylate-able residue was found there instead, this was called a mismatch; for example, the Reactome annotation on Protein X indicates a phosphorylation on a threonine at location 362 whereas UniProt has a tyrosine at position 362. Finally, if the annotated residue was neither a match or a mismatch, the sequence was searched to find the closest exact match and the offset recorded. Once again using the example above, in Reactome another annotation on Protein X indicates a phosphorylation on a serine at position 495, whereas UniProt lists the closest serine to that location is actually at position 490; this would be counted as an offset of 5. It should be noted that if there was no match to the canonical residue all isoforms were then searched for the same criteria (match, mismatch and offset). Annotated modifications where the position is larger than the canonical sequence length, or where there is no position annotated at all, are considered to have not matched and no mismatch or offset is searched for.

### Experimental data

We used phosphoproteomics data from 191 publications across 484 conditions obtained downloaded from the qPhos repository to assess the coverage of phosphoproteomics data by Reactome, PhosphoSitePlus, HPRD, BioGRID and SIGNOR. Data was downloaded from the qPhos download portal in January 2019. Each modification in qPhos was associated with a PubMed ID (PMID) and each PMID was tagged with metadata, such as the ‘sample’ (cell line) or condition the experiments were performed in. We subset the phosphosites by cell line and tissue type. Only phosphosites (and inherently PMID’s) tagged with a single ‘sample’ type (such as MCF-7 or HeLa) were analysed (in cases where there were multiple conditions it was impossible to discern which modification came from which sample).

### Software and data availability

The source code and precompiled binaries are available from github (https://github.com/DavisLaboratory/Db_Compare). The software is compatible with Linux and MacOS operating systems and has been tested on both. The software requires Java version 8, Python 3 and R 3.6. Installation of these and dependencies is managed through the Anaconda package management system; a full list is available in the github repository along with installation instructions.

## Results

### What proportion of the proteome is covered by different pathway databases?

The full extent of human phosphorylation is unknown and is an active field of research today. One study focused on profiling a single cell line identified approximately 75% of proteins to be phosphorylated (Sharma et al., 2014). Yet, many phosphoproteomics studies perform pathway level analysis at the protein level. This is due to the fact that many databases do not offer phosphosite level mapping. This motivated us to first determine the proteome coverage (and by proxy the coverage of proteins which may potentially be phosphorylated) by each database. This was done in order to provide context around their phosphoproteomic coverage as well as to assess their individual and combined potential for analyzing phosphoproteomic datasets. To do this, Reactome, WikiPathways, KEGG, HPRD, BioGRID, SIGNOR and PhosphoSitePlus were downloaded and their protein lists extracted. We normalised all protein UniProt identifiers to the January 2021 version of UniProt as explained in the methods. Because our focus is on resources that capture signalling reactions, throughout the analysis we used the subset of PhosphoSitePlus that contained kinase-substrate pairs (14,159 pairs) (henceforth referred to as PhosphoSitePlus (K-S) and PSP (K-S) in figures) and did not compare to phosphorylation events with no known kinase (231,645 events).

**Figure 1:**
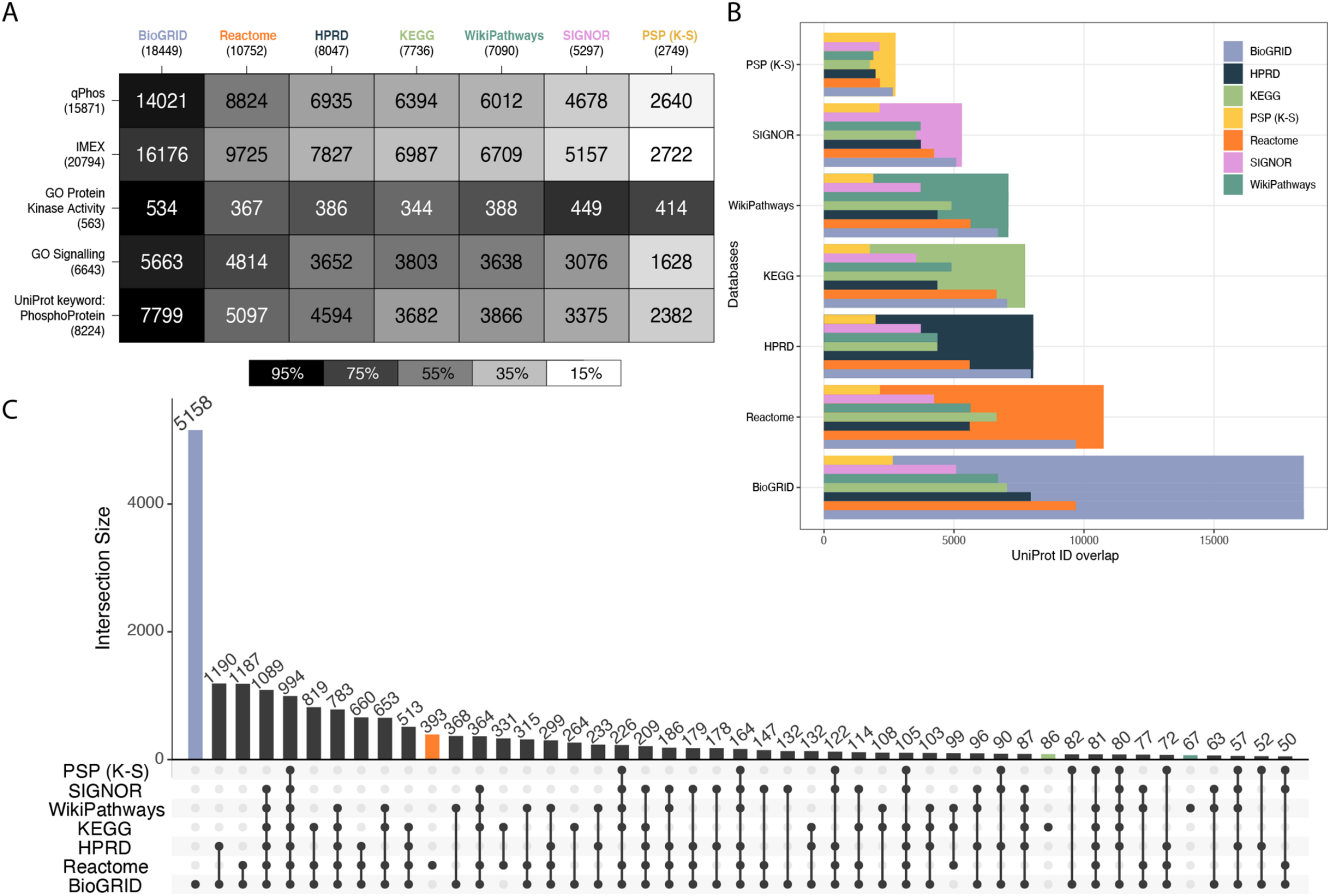
Cross database comparison of proteins. (A) A heatmap of proteins comparing knowledgebases with reference datasets, where each cell is a pairwise intersection, the number represents the number of proteins found in both the reference dataset (vertical axis) and in the signalling database (horizontal axis); the total number of proteins per database is listed under the database name and the cell shade represents the proportion of coverage of each reference dataset (vertical axis); the darker the colour the higher the coverage of that dataset by its paired signalling database. (B) Each pairwise protein level database intersection displayed where the largest bar represents the total number of proteins in that respective database and the inset bars capture the number of overlapping entities from each resource. (C) An UpSet plot depicting intersections of proteins between the databases Reactome, HPRD, KEGG, WikiPathways, PhosphoSitePlus, BioGRID and SIGNOR; vertical bars indicate the number of UniProt ids found in the intersection of the databases listed with a black dot underneath. Coloured bars capture the number of proteins unique to a resource.

To characterize each database’s coverage of the proteome, a collection of reference knowledgebases and datasets were chosen for comparison (Fig. 1A). This set included qPhos, IMEx (Orchard et al., 2012), the Gene Ontology Cell Signalling Category (GO:0023052), the Gene Ontology Protein Kinase Activity Category (GO:0004672) (Ashburner et al., 2000) and the subset of UniProt annotated with the term ‘Phosphoprotein’ (KW-0597). The intersections between each reference dataset and each signalling database were then taken. Intersections with qPhos are expected to represent each knowledgebase’s coverage of phosphorylate-able proteins, while the intersections with IMEX are expected to represent each knowledgebase’s coverage of global interacting proteins, many of which have potential to be phosphorylated. We found BioGRID to have the highest coverage of each reference dataset and PhosphoSitePlus (K-S) had the lowest (barring the GO Protein Kinase Activity category where it had the 3rd highest coverage). Also of interest is the pattern of decreasing coverage of the all reference datasets across the signalling databases excepting the GO Protein Kinase Activity category. We see that BioGRID contains the most known human kinases, followed by SIGNOR, PhosphoSitePlus (K-S), WikiPathways, HPRD, Reactome and finally KEGG. Taken together these results point to a relative ranking for the analysis of data when mapping on the protein level, where you can expect BioGRID to have a high coverage of a given dataset, followed by Reactome, HPRD, KEGG, WikiPathways, SIGNOR and PhosphoSitePlus (K-S) but a differing coverage of the known human kinome. The same order was found when looking at each database’s coverage of SwissProt as well at the entirety of UniProt (Fig. S3, Supporting Information).

To explore the distinctions between each database’s proteome coverage we compared the database protein sets with each other using an UpSet plot (Conway, Lex & Gehlenborg, 2017). Overlaps of less than 50 were excluded, the entire UpSet plot can be found in Fig. S4, Supporting information. Examining the proteins common to all databases, we see the global intersection is just 5% of the total 19,816 unique proteins held across them (Fig. 1C), indicating a marginal global overlap. Individually, BioGRID consistently has the highest overlap with all other databases (Fig. 1B). Although the global intersection is small, the proportion of each database that is unique to itself ranges from only 2.5–20% (Fig. S4). We see that once again BioGRID consistently has the highest coverage of the other databases followed by Reactome in every database. WikiPathways and KEGG appear to have similar coverage of each other database. Finally, SIGNOR consistently has the 6th highest coverage (in all databases excepting PhosphoSitePlus (K-S)) and PhosphoSitePlus (K-S) the lowest coverage. The balance of proteins found in other databases vs. found only in a single database is an indication of potential information gain and novel insight when choosing which database and type to use. The set differences of each pair were also investigated to understand the scope of uniqueness of each reference dataset and signalling knowledgebase (Fig. S5, Supporting Information). Lastly, of note, PhosphoSitePlus (K-S) always maintains the lowest coverage of the other databases yet is the highest covered itself. This comparison is important as PhosphoSitePlus contains the most comprehensive known K-S network and each database’s relative coverage provides further evidence to its ability to connect phosphorylation signalling to a greater context.

As a potential consequence of the differing curation practices each database has adhered to, each database has tended to focus on a different area of biology. Of the number of proteins unique to each database, BioGRID had the most at 5,158, followed by Reactome with 393, KEGG and WikiPathways with 86 and 67 respectively, and then SIGNOR, HPRD and PhosphoSitePlus (K-S) all with less than 25 (Fig. 1C, coloured bars, Fig. S4 coloured bars). This alludes to the potential impact of database choice on conclusions drawn when performing pathway enrichment analysis. To explore this further, we performed an over enrichment analysis using the GO Molecular Function category on the proteins found to be unique to each database (Fig. S6, Supporting Information). We found that the proteins unique to Reactome were mostly involved in transporter activity. This could be because it is the only database in our comparison with abundant subcellular location annotations. BioGRID’s unique proteins were enriched for demethylase activity. WikiPathways was enriched for receptor activity. HPRD, SIGNOR, PhosphoSitePlus (K-S) and KEGG’s unique proteins did not produce any enrichment.

### What proportion of the phospho-proteome is covered by different pathway databases?

We sought to address the question of the level of phosphoproteomic coverage of each database. None of the included databases (excepting PhosphoSitePlus (K-S)) have the ability to input a list of phosphosites and have them mapped on the phosphoproteomic level. To do this, all phosphorylation annotations were taken from each database where possible; neither KEGG nor WikiPathways were included in this section of the analysis as modification annotations in these databases are inaccessible.

**Figure 2:**
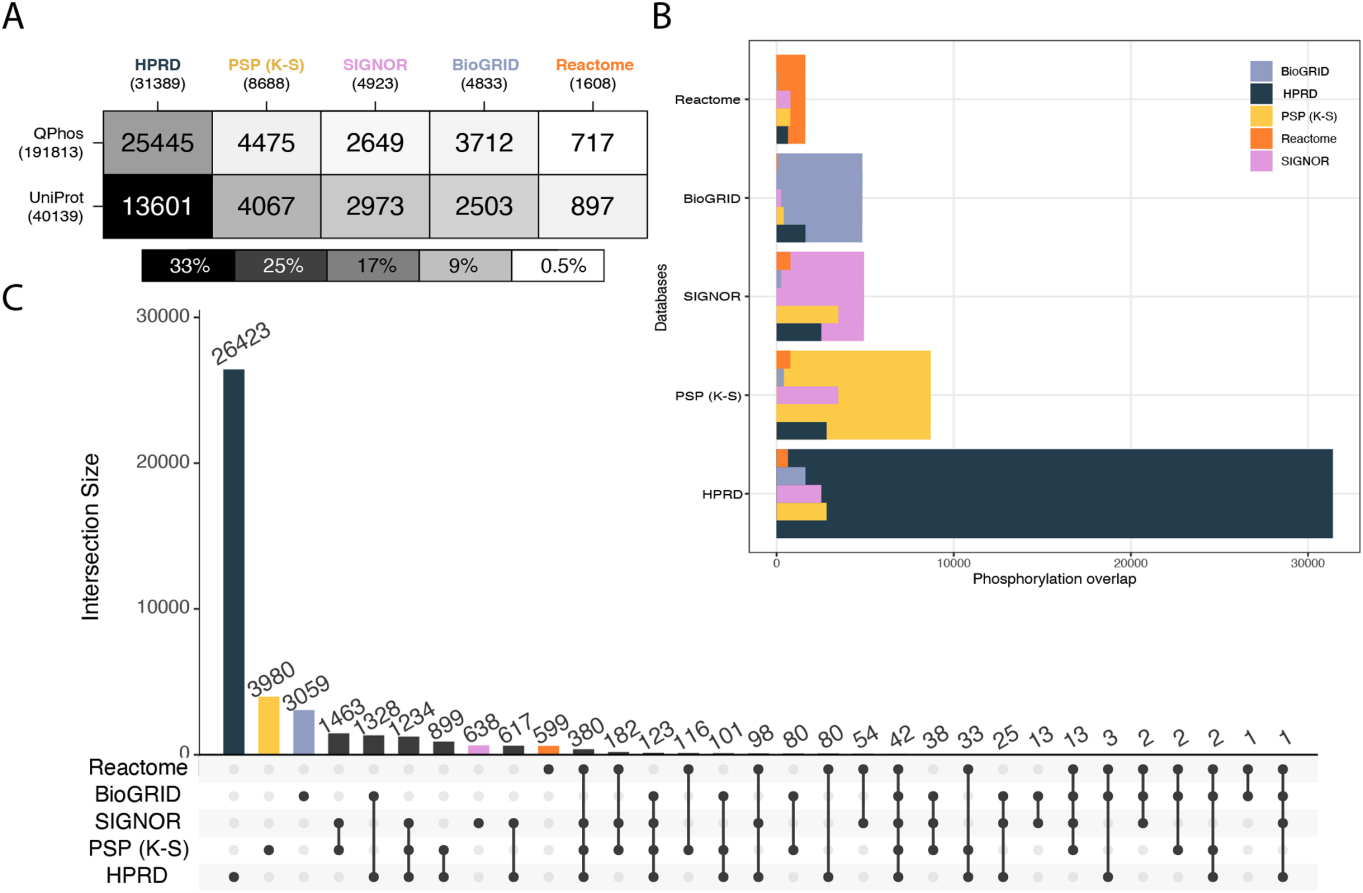
Cross database comparison of phosphorylation annotations. (A) A heatmap where each cell is a pairwise intersection, the number represents the phosphorylations annotated in both the reference dataset (vertical axis) and in the signalling database (horizontal axis); total number of phosphorylations per database is listed under the database name; cell shade represents the proportion of coverage of each reference dataset, the darker the colour the higher the coverage of that dataset by its paired signalling database. (B) Each pairwise phosphorylation level database intersection displayed where the largest bar represents the total number of phosphorylations in that respective database and the inset bars capture the number of overlapping entities from each resource. (C) An UpSet plot depicting all intersections of phosphorylations annotated in BioGRID, HPRD, PhosphoSitePlus, SIGNOR and Reactome; vertical bars indicate the number of phosphorylation annotations found in the intersection of the databases listed with a black dot underneath. Coloured bars capture the number of proteins unique to a resource.

In the first level of database characterization of coverage of the phosphoproteome, the databases of interest were compared to qPhos’ phosphorylation annotations as well as UniProt’s. qPhos contains 39,652 less phosphorylation annotations than the entirety of PhosphoSitePlus. However, qPhos was chosen as the authority on experimentally derived phosphorylations in this analysis, to avoid the self-comparison that would result from using a component of PhosphoSitePlus in evaluating its own utility. Pairwise Intersections were taken between each signalling knowledgebase and each reference dataset (Fig. 2A). Once again, we see a relatively consistent ranking of the databases, but in a differing order than the protein level. Here, HPRD has the highest coverage of qPhos’ phosphorylations, followed by PhosphoSitePlus (K-S), BioGRID, SIGNOR and then Reactome. A slightly different pattern occurs in the coverage of UniProt phosphorylations where the pattern follows the number of phosphosites in each database. Although HPRD has the highest number of phosphorylations annotated in the database category (with 43% of its phosphosites also recorded in UniProt) its coverage only represents 33% of all phosphosites recorded in UniProt. qPhos has almost 5× more phosphorylation annotations than UniProt, and we see that HPRD has the highest overlap with 81% of its phosphorylation events found in qPhos, but still only representing 13% of qPhos’ total. Next is PhosphoSitePlus (K-S), SIGNOR and then BioGRID with 52%, 54% and 77% of their phosphosites respectively in common with qPhos. Yet, each database only covers a small proportion of qPhos at less than 3% for all three. Reactome has the smallest coverage of qPhos at 0.3% but this accounts for 46% of its own database. This means that approximately half of each of the latter 4 databases or more appear in global experimental datasets, but only a tiny proportion of the global experimental data can be interrogated through these knowledgebases. Taken together, this points to the disproportionate amount of data available and the need for tools to assign functional annotations. Added to the knowledge that only 20% of kinases are associated with 87% of currently annotated phosphosites (Needham et al., 2019), there is clear need to start to explore lesser known kinases and their contribution to cellular regulation.

Unlike on the proteomic level we see is no clear pattern in the overlap between databases (Fig. 2B). We see that the levels of uniqueness are much higher. The varying levels of overlap once more indicate the potential new information to be gained when mapping to a database. Keeping in mind that the subset of phosphosites used here from PhosphoSitePlus (K-S) all have a kinase or phosphatase linked to the feature. Though mapping events at the phosphorylation level is not yet supported by these databases, manual inspection of proteins identified in a study may reveal the presence of particular phosphorylation events of interest and their associated reactions, which may be then further explored. This analysis (Fig. 2) can be used as a guide to determining relative likelihood of finding a phosphorylation of interest in each database and the likely level of information that will be associated with it.

When looking at the inter-database coverage we see that the number of phosphosites found in all 5 databases is only 42, a mere 0.1% of the total 41,629 unique phosphorylation annotations across these databases (Fig. 2C) meaning each database covers a very different portion of the phosphoproteome. HPRD has 84% of its phosphosites unique to itself while PhosphoSitePlus (K-S) has 46%, followed closely by BioGRID at 63% Reactome at 37% and lastly SIGNOR at 13%. This is a clear indication of the vastness of the phosphoproteome and further the importance of database choice when it comes to analysing phosphoproteomic data. When looking at the number of unique phosphorylations belonging to each database, HPRD possesses the most at 26,423, PhosphoSitePlus (K-S) at 3,980, followed by BioGRID at 3,059, SIGNOR with 638 and Reactome with 599. The large number of unique phosphosites found in HPRD indicate its dedication to the curation of novel phosphosites (Goel et al., 2012). The set differences of each pair were also investigated on the phosphorylation level to understand the scope of uniqueness of each reference dataset and signalling knowledgebase (Fig. S7, Supporting Information).

### Exploring the consistency between annotated phosphorylated residues and UniProt sequences

A major issue often found in phosphoproteomics is the incorrect assignment of substrates to kinases. This phenomenon is detailed by Humphrey et al. where they described that the correction of low-quality annotations is more difficult than an initial assignment due to the amount of evidence needed to refute prior knowledge. A major compounding factor to this problem is the lack of consistency in substrate phosphorylation annotations across databases. These mis-annotations can often be propagated by both human curators as well as text mining systems if left unchecked, contributing to the incorrect kinase assignments (Bachman, Gyori & Sorger, 2019). To explore this aspect of the problem we decided to test the consistency, or accuracy of the identity of annotated phosphorylated residues against canonical protein sequences found in UniProt. For example, if a database recorded a protein to have a phosphorylation of a serine on residue 456, we wanted to know if a serine would actually be found in the canonical (or isoform) protein sequence at position 456 (described in more detail in methods). Only phosphorylation sites annotated on the canonical protein sequence of a gene were included in Table 1. A match in an isoform was only searched for if a match was not found in the canonical sequence. Further, although a number of phosphorylations have been found on Histidine (Fuhs & Hunter, 2017), here we limited phosphorylate-able residues to Serine, Tyrosine and Threonine.

**Table 1:** Comparing annotated modification position consistency. Statistics of the modified residues annotated in each resource. Number of Modified residues corresponds to the number of unique phosphorylated residues in a protein sequence (only a representative protein per gene is analysed). Percent of consistent residues indicates the percent of annotated modified residues that were consistent with the amino acid residue listed in UniProt at that location. Isoform matches refer to the number of modified residues where the annotated residue was consistent with a protein isoform sequence rather than the canonical protein sequence. Mismatch residue count contains the number of phosphorylated sites where a different phosphorylate-able residue was found in the UniProt sequence rather than the one listed in the named database. The closest exact match offset is the number of modified amino acids found at an offset to the originally annotated modified position.

	qPhos		PSP (K-S)		PSP-full		HPRD		BioGRID		Reactome		SIGNOR	
Number of modified Residues	191,813		8,688		231,465		31,385		4,833		1,608		4,923	
Percent of consistent residues*	189,928 (99.0%)		8,656 (99.6%)		230,694 (99.7%)		30,885 (98.4%)		4,272 (89.5%)		1,250 (77.7%)		4,840 (98.5%)	
Isoform matches	657		3		260		202		151		37		33	
Mismatch residue count	192		2		64		43		47		44		7	
Closest exact match offset	1	424	1	26	1	186	1	144	1	97	1	36	1	14
	2	248	2	2	2	102	2	81	2	64	2	23	2	13
	3	129	3	1	3	63	3	36	3	42	3	14	3	2
	+	792	+	1	+	336	+	152	+	240	+	134	+	36

**Note:**

* Note the percent of consistent residues does not include isoform matches.

Mismatched sites can typically occur because their positions were annotated on post-translationally cleaved proteins, non-canonical isoforms, or on non-human orthologues (Bachman, Gyori & Sorger, 2019). It is likely that an offset of −1 is due to initial methionine cleavage. Offsets of larger size and differing direction are likely to be caused by other issues. The closest matching residue may not be the modified residue. In general, resolving inconsistencies like these will require individual investigation, however automated approaches have been developed that may address the most common sources (Bachman, Gyori & Sorger, 2019). Alternatively, allowing for small offsets (<2) may improve mapping by accounting for slight inconsistencies in annotations (Babur et al., 2018). We report all offsets here for completeness.

We found that the entirety of the PhosphoSitePlus database had the highest sequence consistency with UniProt canonical sequences at 99.7%. We found 260 cases where a match was found in an isoform rather than the canonical sequence, mostly owing to discrepancies with UniProt nomenclature (Table 1). In addition, we found 64 cases where the wrong phosphorylate-able residue was found, labelled a mismatch. Further, looking at PhosphoSitePlus’ K-S subnetwork, we see that it is almost the same at 99.6% consistent. We discovered only three phosphosites were found in isoforms as well as only 26 cases where the annotated residue was found next to the annotated location (i.e. offset by one residue). Next looking at qPhos we see a 99% percent consistency followed by SIGNOR with 98.5% consistency. So far each of these databases has shown exceptional curation practices in gathering phosphoproteomic data. Turning to HPRD we found overall phosphosite consistency to be 98.4% in relation to UniProt canonical sequences, followed by BioGRID with a phosphosite consistency of only 89.5%. Finally, we see Reactome which is 77.7% consistent. This lower consistency may be attributed to the manual curation practices of Reactome, or imprecise recording of isoforms in the literature curated for the database.

### How well do databases cover experimental phosphoproteomic data?

Using phosphoproteomics to interrogate how cells sense and respond to their environment can lead to important insights into biological mechanisms behind these changes. Searching ‘phosphoproteomics’ in PubMed reveals over 3,500 results as well as depicting a steady rise in publications related to the search term over the last 20 years with 456 new publications in 2019 alone (Fig. S8, Supporting Information). As a result of the rise in interest and potential of phosphoproteomics, a number of databases have attempted to assemble the vast amounts of phosphoproteomic data being generated. As seen above, qPhos is one such database that provides curated phosphoproteomics datasets across 191 publications under a wide range of conditions. In this analysis we compared data from these cells with each knowledgebase to establish what proportion of the measurements from each cell type would map onto the knowledge contained in pathway databases. To pose the question another way, we sought to establish the coverage of pathway databases over phosphoproteomics experiments. Here we present results for the top six cell lines, ranked by the number of publications archived in qPhos (Table S3, Supporting Information). The full results are presented in Tables S4 and S5, Supporting Information.

**Figure 3:**
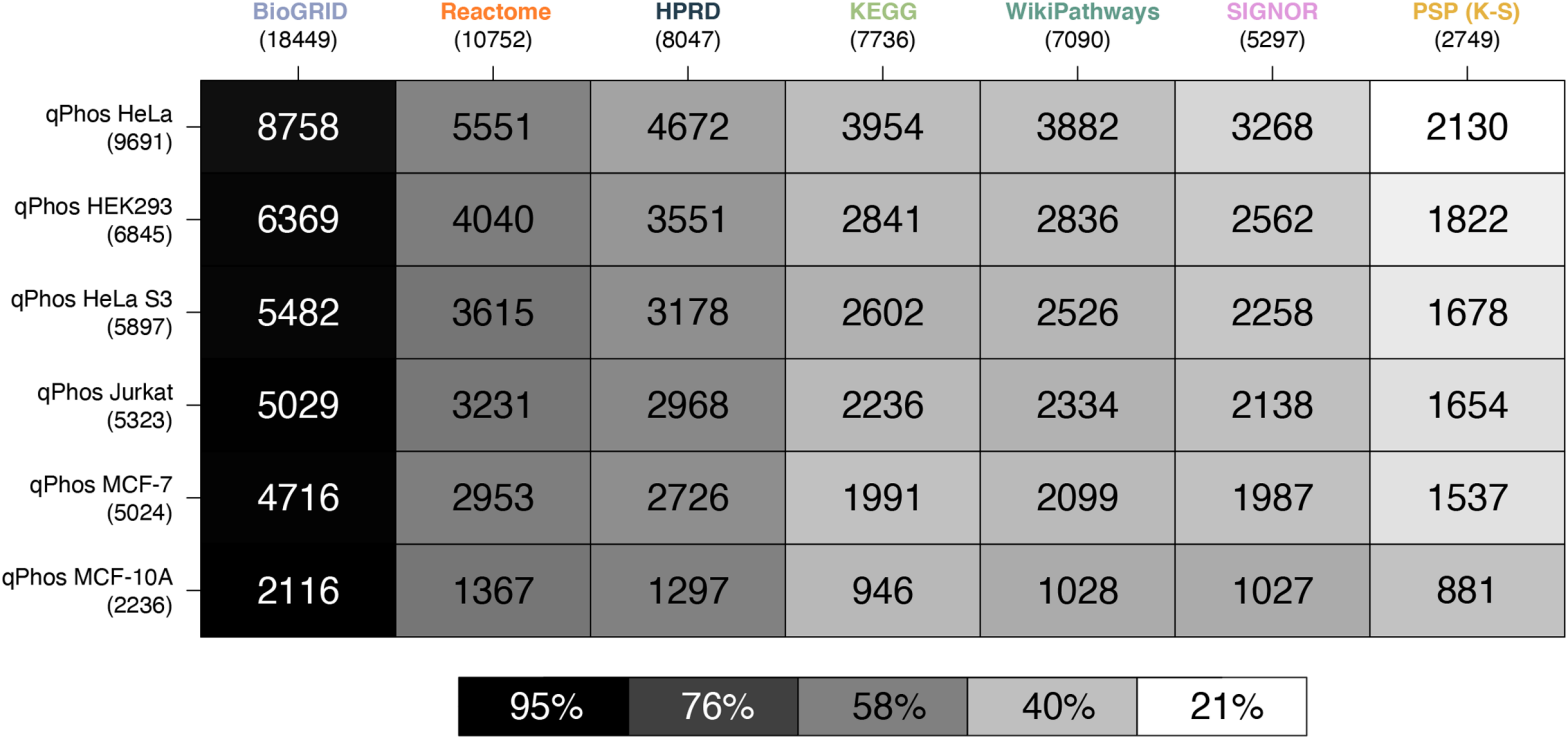
Heatmap of proteomic coverage by databases. A heatmap where each cell represents the pairwise intersection of proteins between the pathway databases being analysed (listed on the horizontal axis), and all proteins found in qPhos that had been tagged with the corresponding cell line (listed on the vertical axis); total number of proteins per database are listed under the database name; shade of each cell refers to the proportion of proteins per cell line that intersect with each pathway knowledgebase.

Consistent with the coverage analysis we performed against the full proteome and the phosphoproteome, we see the same relative ordering in the databases in terms of coverage over experimental data. When compared at the protein level BioGRID has the highest coverage, followed by Reactome, HPRD, KEGG, WikiPathways, SIGNOR and lastly PhosphoSitePlus (K-S) (Fig. 3). Noting the order in which the cell lines are covered by each database, we see that coverage is highest for HELA cells across all databases, followed by HEK293, HeLa S3, Jurkat, MCF-7 and finally MCF-10. This order follows the number of proteins identified in each cell line in a decreasing order, which likely explains the pattern.

**Figure 4:**
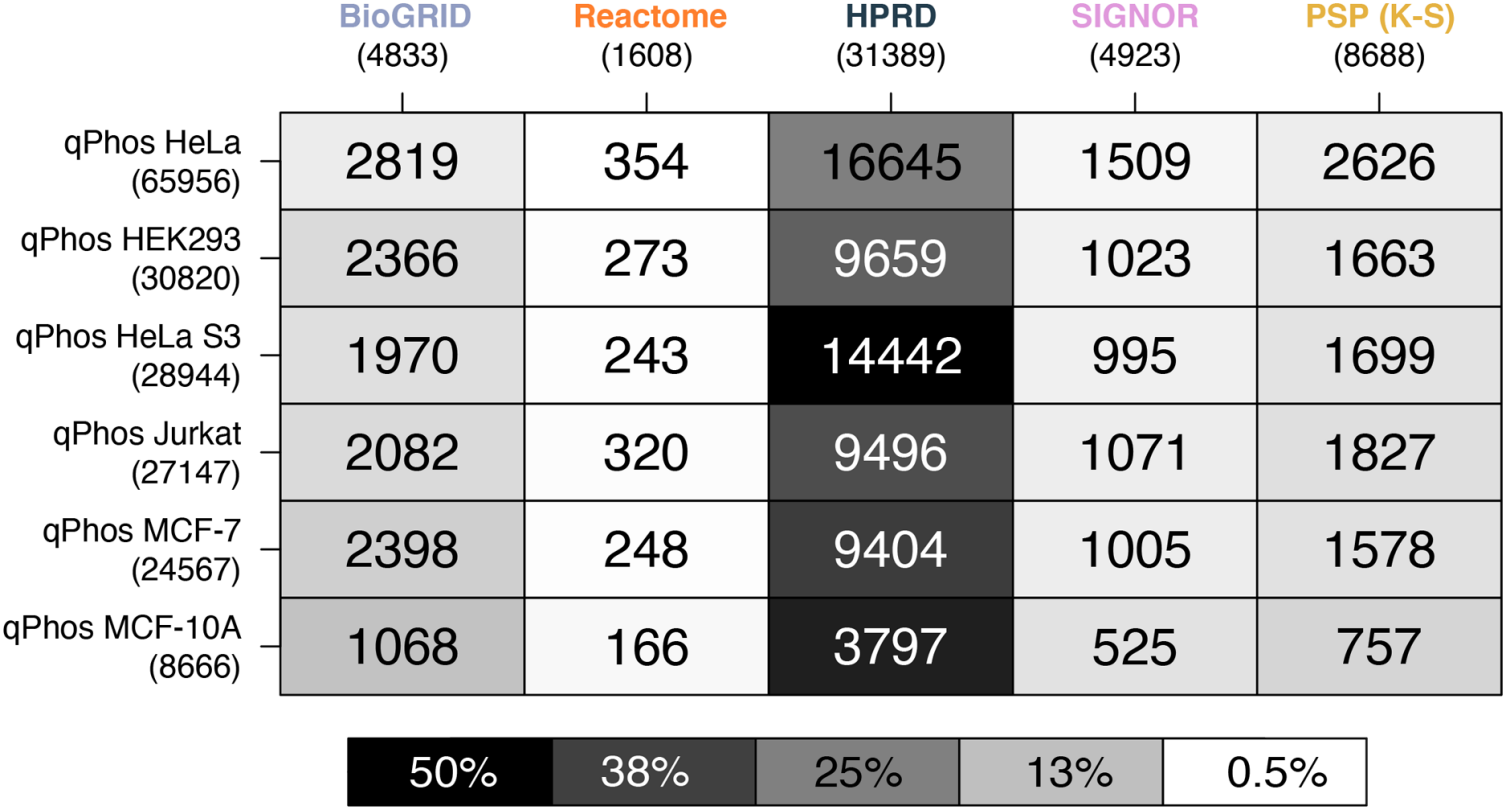
Heatmap of phosphosite coverage by databases. A heatmap where each cell is a pairwise intersection, the number represents the phosphorylations between the pathway databases being analysed (listed on the horizontal axis), and all phosphorylations found in qPhos that had been tagged with the corresponding cell line (listed on the vertical axis); total number of phosphorylations per database is listed under the database name; the shade of each cell refers to the proportion of phosphorylations per cell line that intersects with each pathway knowledgebases annotated phosphorylation.

When comparing coverage of individual phosphorylation events, we see that HPRD has the highest coverage, followed by, BioGRID, PhosphoSitePlus (K-S), SIGNOR and finally Reactome (Fig. 4). In contrast to findings on the proteomic level, when looking at the phosphorylation level data we see a different pattern emerging in the cell line coverage by each database, where once again HeLa is the highest covered but Jurkat, HeLa S3, HEK293 and MCF-7 do not remain consistent and MCF-10 is the lowest covered (Fig. 4). Interestingly this pattern does not follow the number of phosphorylations per cell line. This may be due to the curation practices of each database, potentially highlighting underlying biases towards different common cell lines in experimental data from which pathway knowledge is drawn. These results also demonstrate that the proportion of measured phosphosites for which reaction level information is available is relatively small. For the most commonly measured cell line, HeLa, less than 1% of phosphosites map to annotations in Reactome and attach to known signalling pathways. This small number none-the-less corresponds to 22% of the phosphosites in Reactome. While HeLa phosphosite mapping to PhosphoSitePlus (K-S) is higher at around 4%, this knowledge doesn’t present the same level of mechanistic insight as is captured in Reactome, with functional information limited to K-S interactions. These results (Fig. 4) demonstrate that the vast majority of measured phosphosites lack pathway level annotation.

The contrast in coverage on these two levels by the same databases depict the consequences of generalizing phosphoproteomic level data to the protein level. Global coverage of qPhos can be compared in Figs. 1A and 2A and is reiterated in Figs. 3 and 4’s side-by-side comparison. Through this comparison we see that HPRD has a good coverage of phosphorylate-able proteins and a high coverage of phosphorylations with 56% of its proteins possessing a phosphorylation annotation. Conversely, BioGRID has a high coverage of phosphorylate-able proteins but many fewer phosphorylations when comparing a PPI of similar size (HPRD) with only 19% of its proteins recording a phosphorylation annotation. Reactome has a high level of phosphorylate-able proteins but a low number of phosphorylations translating to 5% of its proteins holding a phosphorylation annotation, while in PhosphoSitePlus (K-S) we see a low number of phosphorylate-able proteins but a high number of phosphorylations (96% of proteins with a phosphorylation annotation). SIGNOR, finally, appears to be on the lower end of proteins but possess a good number of phosphorylation annotations and has 33% of its proteins holding a phosphorylation annotation. This variation in the typical concentration of phosphorylations per protein emphasises the importance of database choice. It also highlights the different strengths of each database should for analyse phosphoproteomics data, as well as the biases that might result from database choice.

## Discussion

We conducted a comprehensive analysis to systematically compare seven widely used knowledgebases, comparing the extent of their coverage over proteome, phosphoproteome and experimental phosphoproteomic data. Recent studies have highlighted the disparity between the amount of phosphosites discovered and the number with known functionality and their impact on cellular signalling (Needham et al., 2019). Here we have attempted to provide novel insights into the relative strengths and weakness of popular literature derived signalling knowledgebases and their comparative suitability for the analysis of phosphoproteomic data.

We found that even though HPRD has not been updated since 2010, it maintains a good coverage of proteins and phosphosites identified in proteomics experiments, as well as a high level of accuracy in the annotated sequence positions of the residues on which phosphorylations are observed. However, as this database is no longer updated it will likely continue to decline in relevance as phosphoproteomic data improves and is disseminated across public databases such as UniProt. The downloadable Protein-Protein Interaction network from HPRD, has a high quantity of proteins and potential interactions, but due to the high-throughput nature of PPI’s, the quality of each datapoint is much lower, making the task of functionally annotating phosphorylations much more difficult. However, the HPRD website contains many phosphosite specific upstream kinases, isoforms as well as many validated low-throughput interactions making it a highly valuable resource still today. HPRD is a great candidate to be used in phosphoproteomic data analysis as a means of hypotheses generation that can be explored further using a more-detailed signalling knowledgebase. However, as previously stated it is no longer actively maintained, and its usefulness is expected to decline over time.

BioGRID had the highest proteomic coverage of the databases assessed but has relatively low phosphoproteome coverage. It also features the second lowest level of consistency of phosphosites with UniProt. BioGRID is actively maintained and being expanded, but in its current form these issues limit its utility in analysing phosphoproteomics data compared to HPRD.

Reactome on the other hand is highly curated with a good coverage of proteomic data, but a much lower coverage of phosphorylation level data especially when combined with a lower consistency between PTM locations and UniProt sequences. Reactome’s high coverage of proteomic data indicates a high mapping potential of phosphorylate-able proteins and although this does not translate to a high mapping potential of phosphorylation sites, it is an important starting point. The few phosphorylation events recorded in Reactome are rich with information. Reactome is the only database in this study to represent proteins in specific phosphorylation states as different entities with differing behaviours, functions and interactions as a direct result of the number and combination of phosphorylations attached. This level of information is critical in gaining functional insights as we know that proteins can be multiply phosphorylated and these phosphorylations can in fact have contradicting or additive effects on a protein’s function (Olsen et al., 2006). However, due to Reactome’s low level of phosphorylation annotations it is not yet comprehensive enough to provide insights to a global phosphoproteomics dataset for more than a very small proportion of the measurements collected in an experiment (see Figs. 2 and 4).

SIGNOR is a database that stores pairwise relationships between biological entities, describing the behaviour of proteins in response to each other (Perfetto et al., 2016). Relationships describe up- or down-regulation with information about the magnitude and mechanism of change. SIGNOR describes 5,297 proteins and 4,923 human phosphorylation annotations, intermediate between a typical PPI database and Reactome. SIGNOR has the highest coverage of phosphosites in PhosphoSitePlus (K-S), indicative of its utility for exploring K-S information in the context of interactions. Consistency with UniProt is high, and the associated information regarding regulators of each individual phosphosite is particularly valuable.

PhosphoSitePlus has a vast amount of information directly relating to phosphoproteomics across species, with most data from human and mouse. It also clearly demonstrates the overload of phosphosites known, in comparison to the amount of phosphosites we biologically understand. Only a subset of this database has the ability to provide mechanistic insights with respect to the kinases likely to be responsible for phosphorylation events. However, the phosphorylations belonging to those proteins were well characterized and very consistent. Like Reactome these phosphosites have a wealth of information attached to them such as regulatory roles and associations with diseases making PhosphoSitePlus the best available resource for inferring kinase activity. However, as this is a specialist database, there is a clear lack of functional information outside of protein modifications, making PhosphoSitePlus a useful tool for database mining but less useful in untangling signalling transduction implications outside of the phosphorylation context.

KEGG and WikiPathways were both found to have a moderate coverage of the proteome but could not be investigated in depth for their use in phosphoproteomics data analysis due to the absence of accessible phosphosite-specific annotation. Nonetheless, today both databases continue to be used in pathway analysis of phosphoproteomic data, largely as a source of gene sets for enrichment type studies (Abe et al., 2020; Park et al., 2015; Kwon et al., 2015; Kawata et al., 2019).

It can be seen in Figs. 3 and 4 that the successful mapping of a phosphoprotein does not necessarily translate to the successful mapping of a phosphosite. In some cases, the protein maps to the knowledgebase, but the knowledgebase has no recorded phosphorylations on that protein, or potentially only a single phosphorylation when a different phosphorylation or even multiple were found on that protein experimentally. The implications of generalizing a phosphoprotein to any unphosphorylated version should be carefully considered. An unphosphorylated protein and protein phosphorylated in a specific manner will most likely behave differently.

When it comes to analysing phosphoproteomic data using general signalling knowledgebases, other factors should also be considered. Firstly, how well does the database cover other types of PTM? PTMs such as ubiquitination and acetylation can have strong cooperative or contradictory effects. For example, an association was found between phosphorylation and acetylation in a genome-reduced *Mycoplasma pneumoniae* (Van Noort et al., 2012) demonstrating that the presence of one modification might facilitate or hinder modification of a closely positioned residue. Another important consideration is the coverage of protein complexes, as they play crucial roles in signal transduction and their formation is often regulated by phosphorylation (Day, Sosale & Lazzara, 2016). Finally, isoforms, which expand the complexity of the proteome, but also present problems for annotation, need to be considered. As shown in Table 1 many modifications are found on non-canonical (alternatively spliced) protein sequences. Isoform specific phosphorylation should be recorded, and the resulting modified isoforms treated as separate entities to their canonical form as they can have a large impact on molecular functions. For example, one study found that Connexin isoform 30 closed upon Protein Kinase C phosphorylation but not connexin isoform 43, completely altering its molecular function (Alstrøm et al., 2015). Of the databases characterized above, Reactome has demonstrated all of these properties making it a valuable starting point for phosphoproteomics functional analysis. While PhosphoSitePlus treats isoforms independently and maintains a repository of PTM’s other than phosphorylation, its kinase-substrate network treats each phosphosite independently, and does not hold any information for multiply phosphorylated proteins nor complexes. HPRD does hold isoforms as well as alternate modifications but in most cases annotates protein–protein interactions as common to all isoforms and modified proteins (Keshava Prasad et al., 2009). Similarly, BioGRID also holds alternate modifications as well as isoforms but does not treat them as separate entities. KEGG holds complexes, and some isoform information (but does not seem to treat them separately), as well as alternate modifications but does not provide the modified residue. Finally, at the time of publication WikiPathways did not have a downloadable BioPax (Demir et al., 2010) file and was unable to be investigated, however it does contain many Reactome pathways which do possess all of the above qualities.

Data saturation is occurring and there is a huge need for bioinformatic analysis to aid in the characterisation of phosphosite function. The use of general signalling knowledgebases has great potential to aid in this endeavour through data browsing, mining and hypothesis generation. No single database analysed in this study emerged as a ‘best’ option, as each had clear strengths and weaknesses. Instead for now these databases should be used in conjunction with each-other to build on the assets that each maintains and makes up for in the others.

## Conclusion

We have used global phosphoproteomics datasets to provide insights into the relative strengths and weaknesses across databases and highlighted general concerns in current phosphoproteomics analysis practices. We found that phosphorylation events in Reactome are rare, but typically come with a subcellular location, as well as interpretable functional annotations for a protein in a specific phosphorylation state (whether that be singly or multi-phosphorylated) leading to potential mechanistic insights. In HPRD phosphorylation events are much more abundant but the downloadable PPI has little information associated with phosphosites. BioGRID possessed few annotated phosphorylation events, meaning it is unlikely that mapping phosphoproteomics data onto this database will yield a high level of successful matches and ultimately helpful insights into phosphoproteomic signalling. SIGNOR on the other hand possessed less phosphorylation annotations but each is highly consistent and holds many other valuable annotations. Many known phosphorylation events can be found in the PhosphoSitePlus database, but most of these do not have K-S annotation. Further, PhosphoSitePlus lacks information on cellular signalling outside the K-S context resulting in limited potential for specific mechanistic insights. The coverage, consistency, number of proteins and number of phosphorylations, as well as the level of detail in each database’s annotations differ greatly. All aspects are important to characterize in order to get a holistic understanding of how each database is capable of characterizing phosphoproteomics data.

## Supplemental Information

10.7717/peerj.11298/supp-1 Supplemental Information 1 Diagram of code workflow.

10.7717/peerj.11298/supp-2 Supplemental Information 2 Neo4j Graph database schema.

10.7717/peerj.11298/supp-3 Supplemental Information 3 A Heatmap of Database Intersections on the Protein Level. Each cell represents the pairwise intersection of proteins between the pathway databases being analysed (listed on the horizontal axis), and all proteins found in SwissProt or UniProt (listed on the vertical axis) with the percent coverage of SwissProt/UniProt listed underneath; total number of proteins per database are listed under the database name; shade of each cell refers to the proportion of proteins per cell line that intersect with each pathway knowledgebase.

10.7717/peerj.11298/supp-4 Supplemental Information 4 Protein level full UpSet plot. An UpSet plot depicting all intersections of proteins between the databases Reactome, HPRD, KEGG, WikiPathways, PhosphoSitePlus, BioGRID and SIGNOR; vertical bars indicate the number of UniProt IDs found in the intersection of the databases listed with a black dot underneath. Coloured bars capture the number of proteins unique to a resource.

10.7717/peerj.11298/supp-5 Supplemental Information 5 Protein level Set Difference Heatmaps. A) a Venn diagram to aid in the interpretation of plots B to C. (B) The set difference of the reference databases and the resource databases; each cell is a pairwise comparison where the number represents the number of proteins found in the reference knowledgebase (vertical axis) and not in the signalling database (horizontal axis), the total number of proteins per database is listed under the database name, the cell shade represents the proportion of total proteins unique to each reference database. (C) The set difference of the resource databases and the reference databases; each cell is a pairwise comparison where the number represents the number of proteins found in the signalling database (horizontal axis) and not in the reference knowledgebase (vertical), the total number of proteins per database is listed under the database name, the shade of the cell represents the proportion of total proteins unique to each reference database.

10.7717/peerj.11298/supp-6 Supplemental Information 6 Over Representation Analysis of UniProt accessions unique to each database, using the Gene Ontology Molecular Function Category. The Gene Ontology Molecular Function category was used. P values reported are less than 0.05. Background used was the unique list of all proteins found in all databases analysed in this manuscript.

10.7717/peerj.11298/supp-7 Supplemental Information 7 Phosphorylation level Set Difference Heatmaps. (A) A Venn diagram to aid in the interpretation of plots B to C. (B) The set difference of the reference databases and the resource databases; each cell represents the number of phosphorylation’s unique to the respective reference database (qPhos or UniProt), the total number of phosphorylation’s per database is listed under the database name, the cell shade represents the proportion of total phosphorylation’s unique to each reference database. (C) the set difference of the resource databases and the reference databases, each cell in this table is the number of phosphorylation’s unique to the respective signalling database (HPRD, PhosphoSitePlus, SIGNOR, BioGRID or Reactome), the shade of the cell represents the proportion of total phosphorylation’s unique to each reference database.

10.7717/peerj.11298/supp-8 Supplemental Information 8 Phosphoproteomics related publications in PubMed. Counts of publications resulting from the search term ‘Phosphoproteomics’ in August 2020.

10.7717/peerj.11298/supp-9 Supplemental Information 9 The 29 original databases considered in this study.

10.7717/peerj.11298/supp-10 Supplemental Information 10 Dates of database access.

10.7717/peerj.11298/supp-11 Supplemental Information 11 Top six qPhos cell lines with the most PubMed ID’s.

10.7717/peerj.11298/supp-12 Supplemental Information 12 Experimental overlap of all qPhos conditions with analysed databases on the proteomic level.

10.7717/peerj.11298/supp-13 Supplemental Information 13 Experimental overlap of all qPhos conditions with analysed databases on the phosphoproteomic level.

## Additional Information and Declarations

### Data Availability

The following information was supplied regarding data availability: The source code and precompiled binaries are available at GitHub: https://github.com/DavisLaboratory/Db_Compare. The software is compatible with Linux and MacOS operating systems and has been tested on both. The software requires Java version 8, Python 3, and R 3.6. Installation of these and dependencies is managed through the Anaconda package management system; a full list is available in the GitHub repository along with installation instructions.
