# Supplementary figures and images for: Measuring pathway database coverage of the phosphoproteome
Source: PeerJ. 2021 May 25;9:e11298. doi: 10.7717/peerj.11298 (PMC8162239; doi:10.7717/peerj.11298)

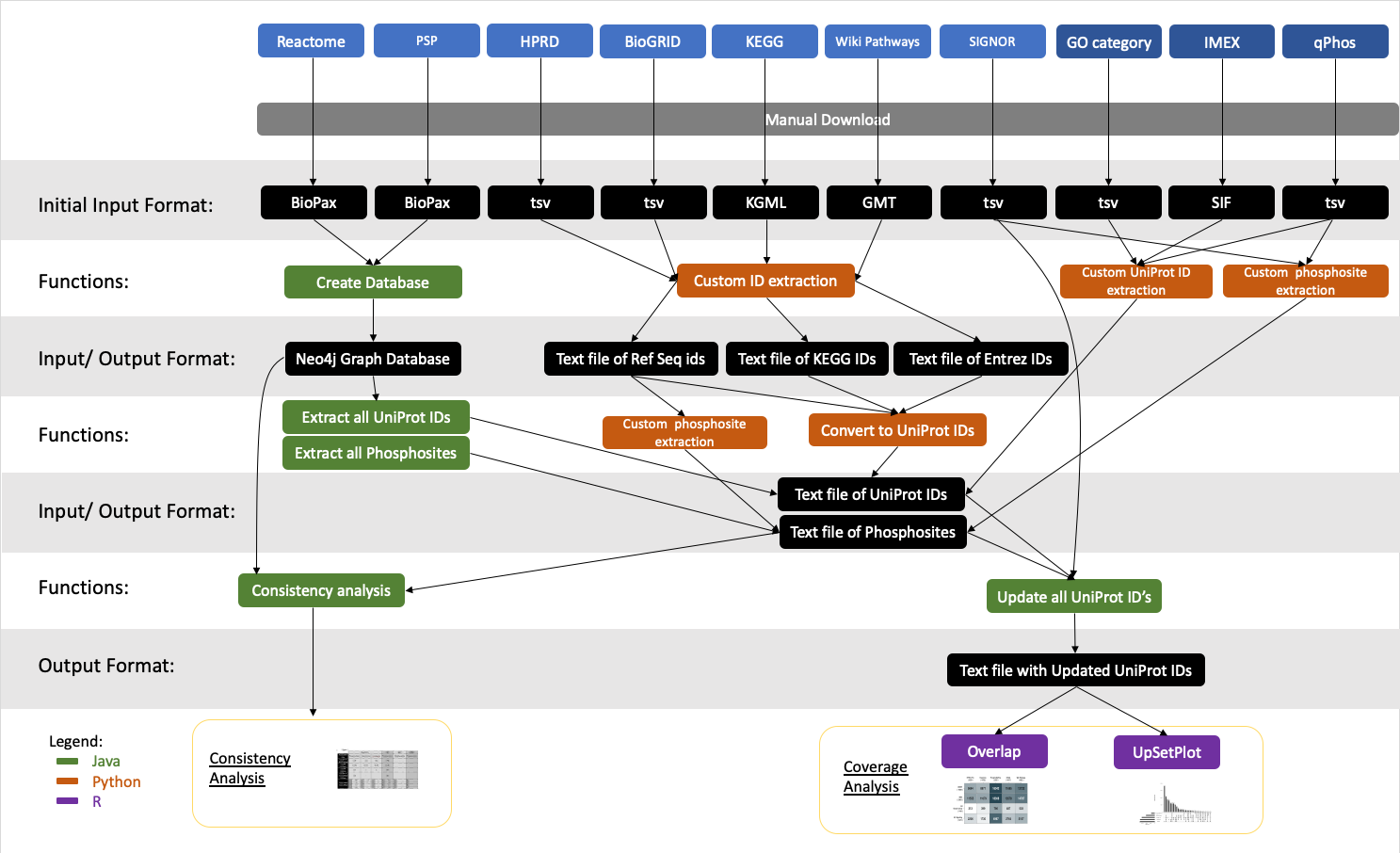

Supplement: Supplemental Information 1 [file peerj-09-11298-s001.png]

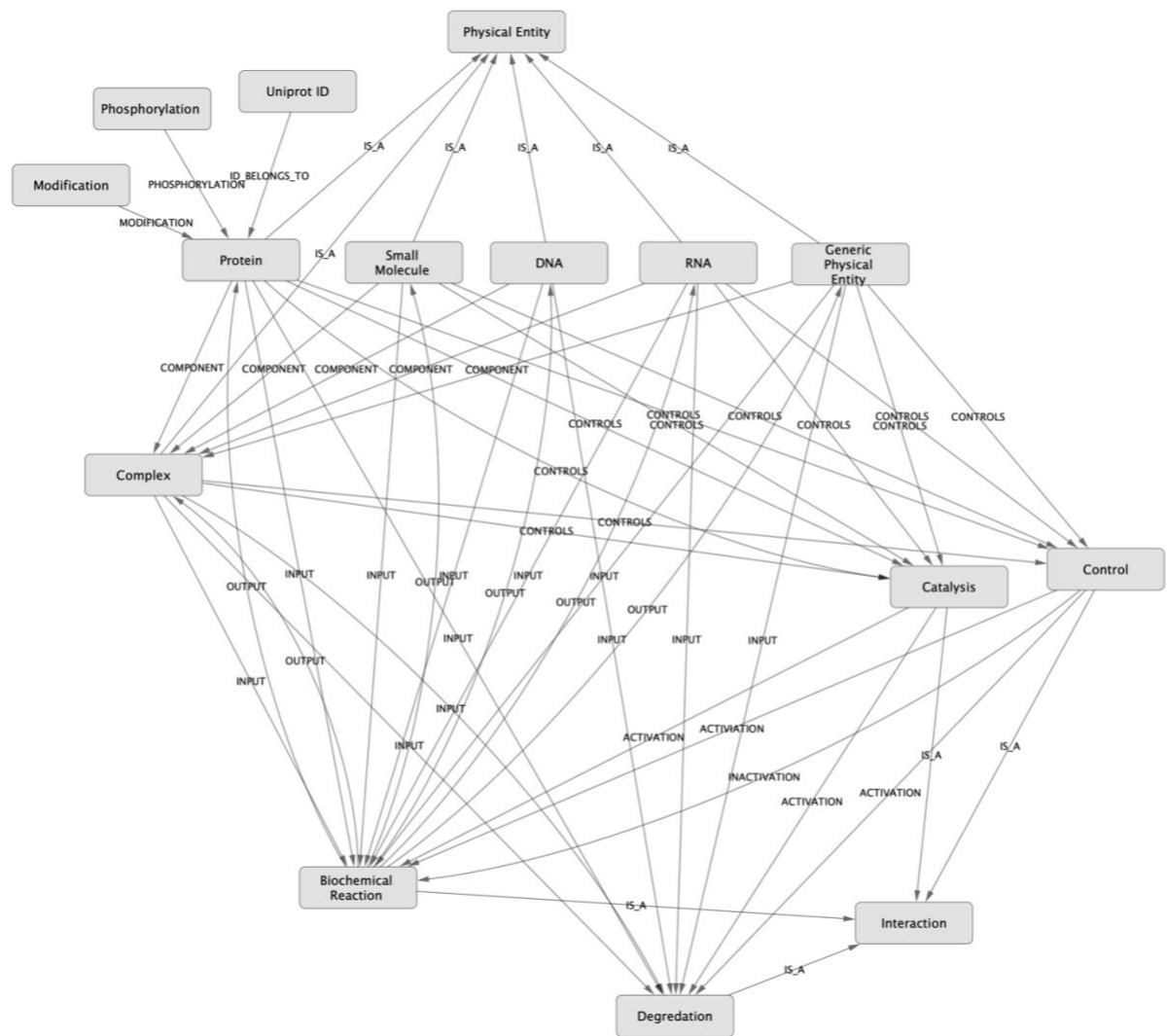

Supplement: Supplemental Information 2 [file peerj-09-11298-s002.pdf]

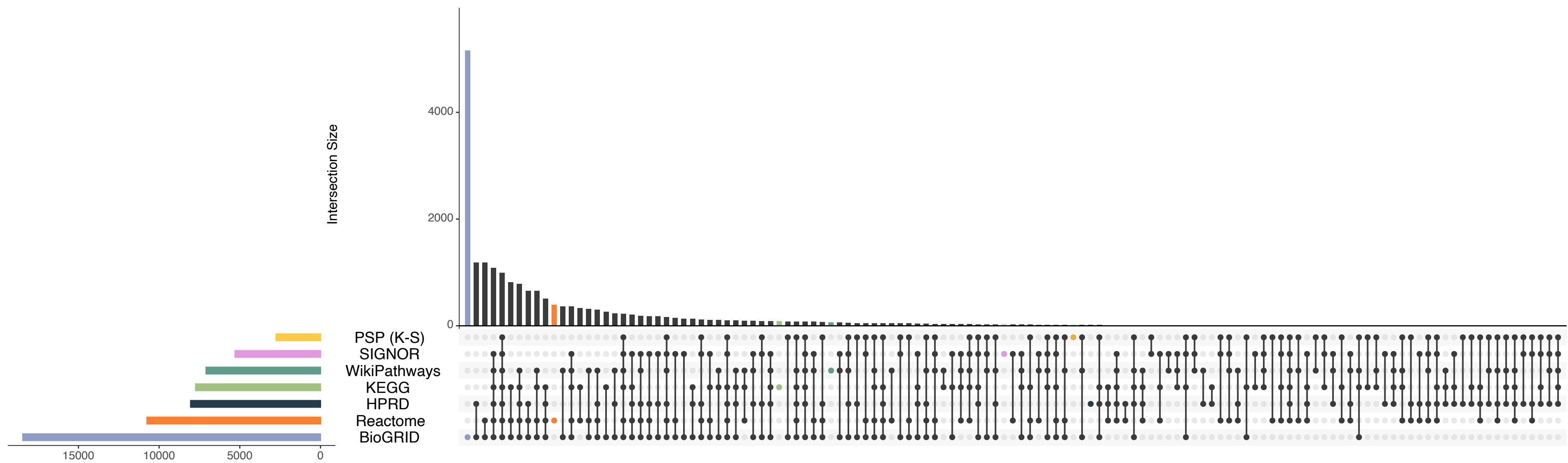

Supplement: Supplemental Information 4 — An UpSet plot depicting all intersections of proteins between the databases Reactome, HPRD, KEGG, WikiPathways, PhosphoSitePlus, BioGRID and SIGNOR; vertical bars indicate the number of UniProt IDs found in the intersection of the databases listed with a black dot underneath. Coloured bars capture the number of proteins unique to a resource. [file peerj-09-11298-s004.pdf]

A)

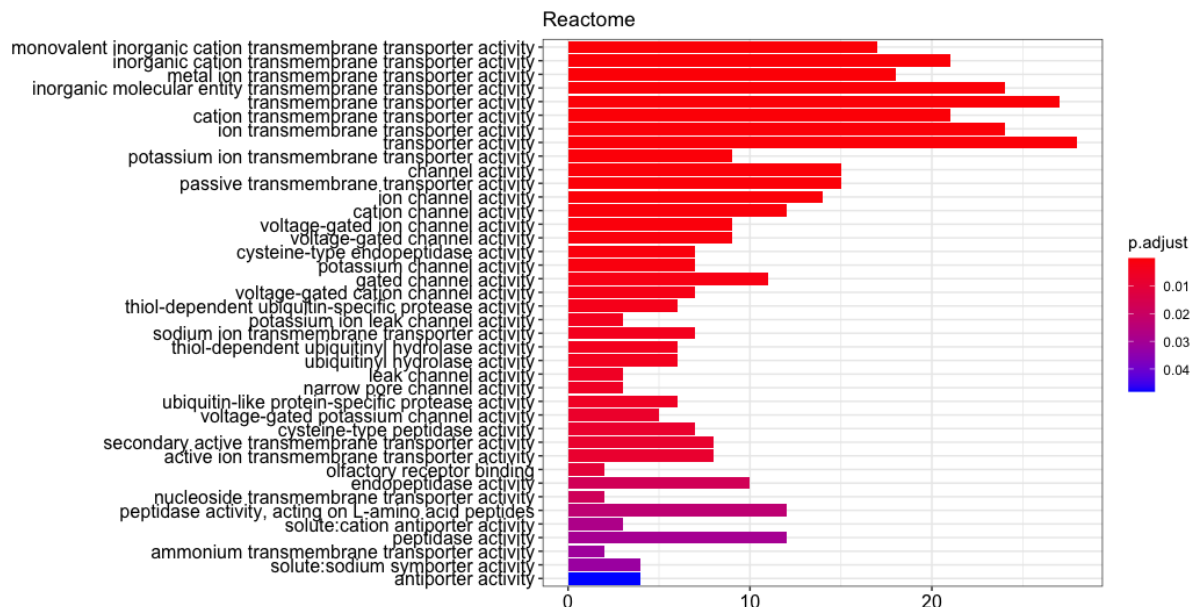

B)

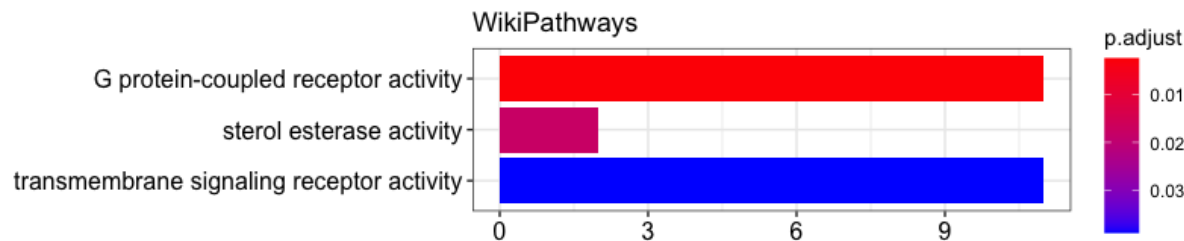

C)

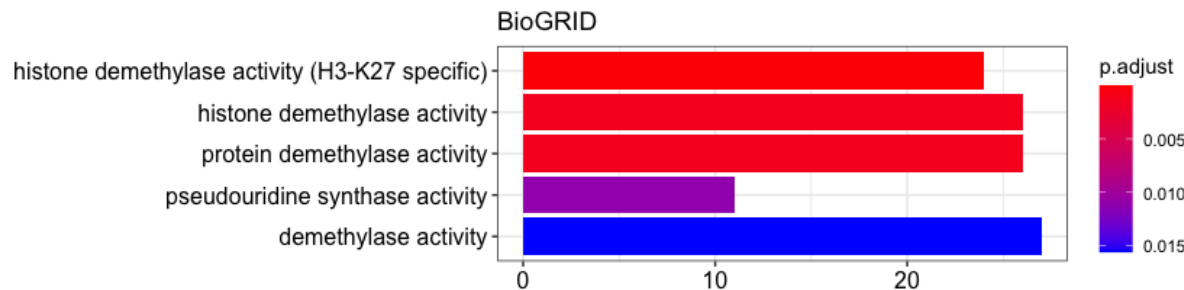

Supplement: Supplemental Information 6 — The Gene Ontology Molecular Function category was used. P values reported are less than 0.05. Background used was the unique list of all proteins found in all databases analysed in this manuscript. [file peerj-09-11298-s006.pdf]

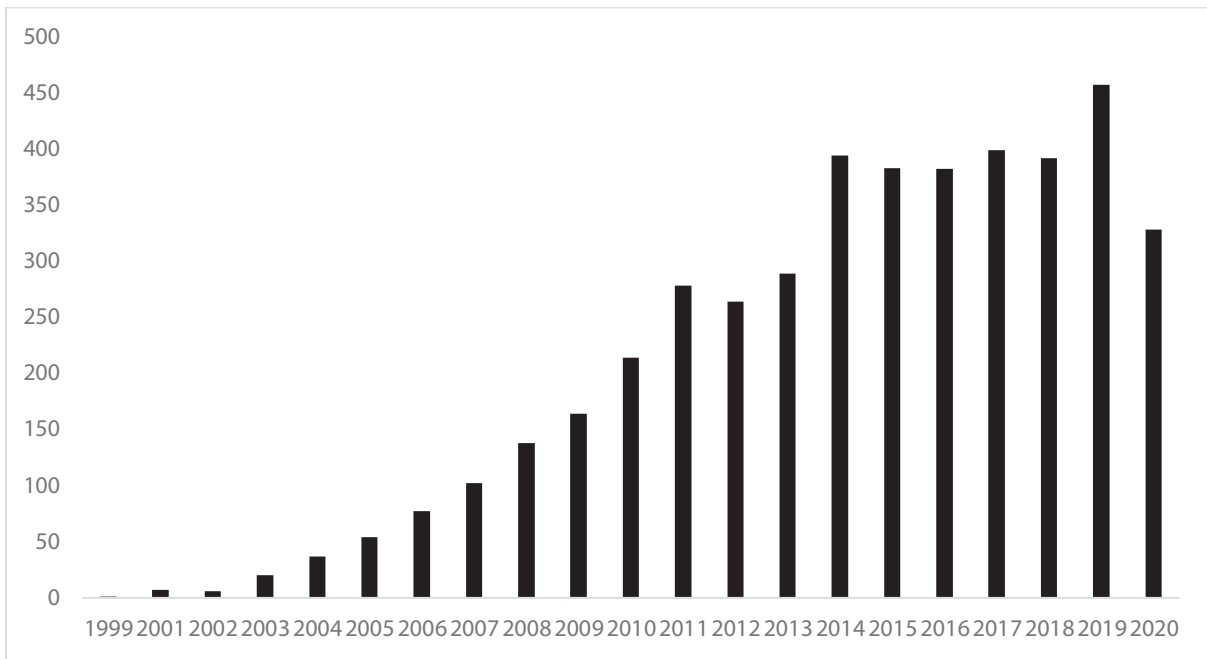

Supplement: Supplemental Information 8 — Counts of publications resulting from the search term ‘Phosphoproteomics’ in August 2020. [file peerj-09-11298-s008.pdf]
