# Supplementary material for: Measuring pathway database coverage of the phosphoproteome
Source: PeerJ. 2021 May 25;9:e11298. doi: 10.7717/peerj.11298 (PMC8162239; doi:10.7717/peerj.11298)
Supplement: Supplemental Information 3 — Each cell represents the pairwise intersection of proteins between the pathway databases being analysed (listed on the horizontal axis), and all proteins found in SwissProt or UniProt (listed on the vertical axis) with the percent coverage of SwissProt/UniProt listed underneath; total number of proteins per database are listed under the database name; shade of each cell refers to the proportion of proteins per cell line that intersect with each pathway knowledgebase. [file peerj-09-11298-s003.pdf]

	HPRD (8047)	BioGRID (18449)	Reactome (10752)	KEGG (7736)	WikiPathways (7090)	PSP (K-S) (2749)
SwissProt (20397)	8018	16917	10717	7690	7063	2745
TrEMBL (194523)	8047	18449	10752	7736	7090	2749
