# Supplementary material for: Measuring pathway database coverage of the phosphoproteome
Source: PeerJ. 2021 May 25;9:e11298. doi: 10.7717/peerj.11298 (PMC8162239; doi:10.7717/peerj.11298)
Supplement: Supplemental Information 5 — A) a Venn diagram to aid in the interpretation of plots B to C. (B) The set difference of the reference databases and the resource databases; each cell is a pairwise comparison where the number represents the number of proteins found in the reference knowledgebase (vertical axis) and not in the signalling database (horizontal axis), the total number of proteins per database is listed under the database name, the cell shade represents the proportion of total proteins unique to each reference database. (C) The set difference of the resource databases and the reference databases; each cell is a pairwise comparison where the number represents the number of proteins found in the signalling database (horizontal axis) and not in the reference knowledgebase (vertical), the total number of proteins per database is listed under the database name, the shade of the cell represents the proportion of total proteins unique to each reference database. [file peerj-09-11298-s005.pdf]

A)

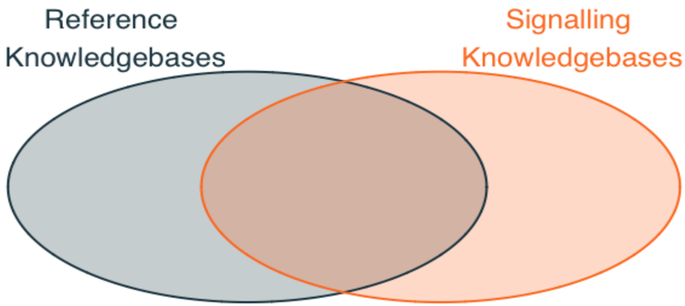

B)

	HPRD (8047)	BioGRID (18449)	Reactome (10752)	KEGG (7736)	WikiPathways (7090)	PhosphoSitePlus (2749)	SIGNOR (5297)
qPhos (15871)	8936	1850	7047	9477	9859	13231	11193
IMEX (20794)	12967	4618	11069	13807	14085	18072	15637
GO Protein Kinase Activity (563)	177	29	196	219	175	149	114
GO Signalling (6643)	2991	980	1829	2840	3005	5015	3567
UniProt keyword: PhosphoProtein (8224)	3630	425	3127	4542	4358	5842	4849

C)

	HPRD (8047)	BioGRID (18449)	Reactome (10752)	KEGG (7736)	WikiPathways (7090)	PhosphoSitePlus (2749)	SIGNOR (5297)
qPhos (15871)	1112	4428	1928	1342	1078	109	619
IMEX (20794)	220	2273	1027	749	381	27	140
GO Protein Kinase Activity (563)	7661	17915	10385	7392	6702	2335	4848
GO Signalling (6643)	4395	12786	5938	3933	3452	1121	2221
UniProt keyword: PhosphoProtein (8224)	3453	10650	5655	4054	3224	367	1922
