# Supplementary material for: Measuring pathway database coverage of the phosphoproteome
Source: PeerJ. 2021 May 25;9:e11298. doi: 10.7717/peerj.11298 (PMC8162239; doi:10.7717/peerj.11298)
Supplement: Supplemental Information 7 — (A) A Venn diagram to aid in the interpretation of plots B to C. (B) The set difference of the reference databases and the resource databases; each cell represents the number of phosphorylation’s unique to the respective reference database (qPhos or UniProt), the total number of phosphorylation’s per database is listed under the database name, the cell shade represents the proportion of total phosphorylation’s unique to each reference database. (C) the set difference of the resource databases and the reference databases, each cell in this table is the number of phosphorylation’s unique to the respective signalling database (HPRD, PhosphoSitePlus, SIGNOR, BioGRID or Reactome), the shade of the cell represents the proportion of total phosphorylation’s unique to each reference database. [file peerj-09-11298-s007.pdf]

A)

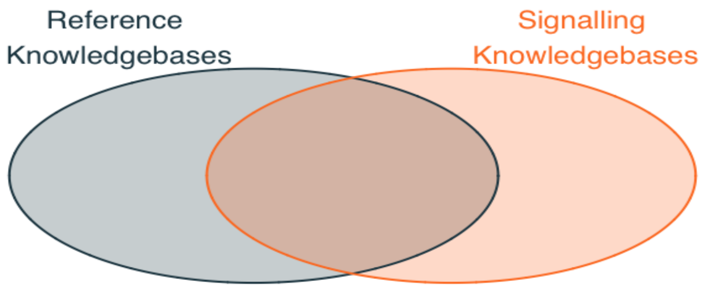

B)

	HPRD (31389)	PhosphoSitePlus (8688)	BioGRID (4833)	Reactome (1608)	SIGNOR (4923)
QPhos (191813)	166368	187338	188101	191096	189164
UniProt (40139)	26528	36062	37626	39232	37156

C)

	HPRD (31389)	PhosphoSitePlus (8688)	BioGRID (4833)	Reactome (1608)	SIGNOR (4923)
QPhos (191813)	5944	4213	1121	891	2274
UniProt (40139)	17788	4621	2330	711	1950
