# Supplementary material for: Measuring pathway database coverage of the phosphoproteome
Source: PeerJ. 2021 May 25;9:e11298. doi: 10.7717/peerj.11298 (PMC8162239; doi:10.7717/peerj.11298)
Supplement: Supplemental Information 9 [file peerj-09-11298-s009.docx]

| **Database name** | **Widely cited?**  **(over 1000)** | **Contains signalling reactions** | **Contains Phospho-rylation**  **sites** | **Price** | **Update scheme** | **Download format** | **Databases it contains** | **Notes** |
| --- | --- | --- | --- | --- | --- | --- | --- | --- |
| **Reactome** | Yes | Yes | Yes | Free | Quarterly | BioPax, tsv,  Neo4j graph database | - | Reactome is a free, open-source, curated and peer-reviewed pathway database. |
| **WikiPathways** | No | Yes | Yes | Free | Constantly | BioPax, gpml | NetPath, Reactome | A database of biological pathways maintained by and for the scientific community |
| **KEGG - public** | Yes | Yes | Yes | Free | Consistently | KGML | - | KEGG is a manually curated resource integrating eighteen databases categorized into systems, genomic, chemical and health information. |
| **Signor** | No | Yes | Yes | Free | Constantly | Tsv, xls, causalTab, gmt | - | Signor 2.0 is a public repository that stores signaling information as binary causal relationships between biological entities. |
| **BioCyc** | No | Yes | unknown | Paid | Consistently | BioPax, SBML | - | BioCyc is a collection of Pathway/Genome Databases (PGDBs), plus software tools for exploring them |
| **INOH** | No | Yes | Yes | Free | Last updated in 2011 | Csv, BioPax | - | No longer exists.  INOH database provides signal transduction pathway data which is well-annotated by the INOH ontology terms. |
| **NetPath** | No | Yes | unknown | Free | Last updated 2010 | Not downloadable | - | NetPath is a manually curated resource of signal transduction pathways in humans. |
| **BioCarta** | No | Yes | Unknown | Free | unknown | Via API | - | No longer exists.  The BioCarta website is an interactive online resource targeted to the life science research community. The content can be divided into three main categories, gene function, proteomic pathways and reagent exchange. |
| **DIP** | Yes | No | No | Free | Last updated in 2017 | XIN, MIF, MIF25, MITAB25 | - | DIP is a database that documents experimentally determined protein-protein interactions. |
| **InnateDb** | No | Yes? | No | Free | Weekly | XML, TAB | IntAct, DIP, MINT, BIND, BioGRID  KEGG, INOH, PID, NetPath, Reactome  (all last drawn from in 2014) | The database captures an improved coverage of the innate immunity interactome by integrating known interactions and pathways from major public databases together with manually-curated data into a centralised resource |
| **IntAct** | Yes | No | No | Free | Monthly | MITAB2.5, MITAB3.0 | - | IntAct provides a freely available, open source database system and analysis tools for molecular interaction data. All interactions are derived from literature curation or direct user submissions and are freely available. |
| **STRING** | Yes | No | No | Free | Consistently | txt | - | The STRING database aims to collect, score and integrate all publicly available sources of protein–protein interaction information, and to complement these with computational predictions |
| **HPRD** | Yes | No | Yes | Free | Last updated in 2010 | tsv | - | The HPRD represents a centralized platform to visually depict and integrate information pertaining to domain architecture, post-translational modifications, interaction networks and disease association for each protein in the human proteome. |
| **BioGRID** | Yes | No | Yes | Free | Monthly | MITAB, TAB | - | BioGRID is a biomedical interaction repository with data compiled through comprehensive curation efforts. |
| **MINT** | No | No | Yes | Free | unknown | MITAB | - | MINT focuses on experimentally verified protein-protein interactions mined from the scientific literature by expert curators. |
| **Mentha** | No | No | Yes | Free | unknown | - | - | No longer exists. |
| **PhosphoSitePlus** | Yes | Yes | Yes | Free | Consistently | BioPax, tsv | - | PhosphoSitePlus provides comprehensive information and tools for the study of protein post-translational modifications (PTMs) including phosphorylation, acetylation, and more. |
| **PhosphoElm** | No | No | Yes | Free | Last updated in 2010 | tsv | - | Phospho.ELM is a database of experimentally verified phosphorylation sites in eukaryotic proteins. |
| **dbPAF** | No | No | Yes | Free | consistently | tsv | PhosphoELM, dbPTM, PHOSIDA,  PhosphositPlus, PhosphoPep,  PhosphoGRID, SysPTM, HPRD,  Uniprot | dbPAF is an online data resource specifically designed for protein phosphorylation in seven eukaryotic species, including H. sapiens. |
| **phosphoGRID** | - | No | Yes | Free | Consistently (in BioGRID) | MITAB, TAB | - | permanently re-directed into the BioGRID website |
| **PhosPhAt** | - | No | Yes | Free | unknown | csv | - | The Arabidopsis Protein Phosphorylation Site Database |
| **PhosphoNET** | - | No | Yes | Free | Last updated 2019 | Not downloadable | - | PhosphoNET is an open-access, online resource developed by Kinexus Bioinformatics Corporation to foster the study of cell signalling systems to advance biomedical research in academia and industry. |
| **PHOSIDA** | No | No | Yes | Free | unknown | Not downloadable | - | Phosida allows the retrieval of phosphorylation, acetylation, and N-glycosylation data of any protein of interest. It lists posttranslational modification sites associated with particular projects and proteomes or, alternatively, displays posttranslational modifications found for any protein or protein group of interest. |
| **PID** | Yes | - | - | - | Never | - | - | No longer exists |
| **BIND** | No | - | - | - | Never | - | - | No longer exists |
| **NDEx** | No | Yes | Yes | Free | Constantly | Can download the NDEx software for private use | Any network in SIF, XGMML, BioPax or OpenBEL format can contained in this database | The NDEx Project provides an open-source framework where scientists and organizations can share, store, manipulate, and publish biological network knowledge. |
| **CORUM** | No | No | No | Free | Current release is 2018 | XML, tsv, jason | - | The CORUM database provides a resource of manually annotated protein complexes from mammalian organisms. |
| **CTD** | No | No | No | Free | Monthly | Csv, tsv, xml | - | CTD is a robust, publicly available database that aims to advance understanding about how environmental exposures affect human health. It provides manually curated information about chemical–gene/protein interactions, chemical–disease and gene–disease relationships. These data are integrated with functional and pathway data to aid in development of hypotheses about the mechanisms underlying environmentally influenced diseases. |
| **Drugbank** | Yes | No | No | Free | Monthly | Xml | - | The knowledge base consists of proprietary authored content describing clinical level information about drugs such as side effects and drug interactions, as well as molecular level data such as chemical structures and what proteins a drug interacts with |
