# Supplementary material for: Measuring pathway database coverage of the phosphoproteome
Source: PeerJ. 2021 May 25;9:e11298. doi: 10.7717/peerj.11298 (PMC8162239; doi:10.7717/peerj.11298)
Supplement: Supplemental Information 10 [file peerj-09-11298-s010.docx]

**Supplementary Table 2: Dates of Database Access.**

| **Database** | **Date downloaded** | **Date updated * CHANGED** |
| --- | --- | --- |
| Reactome | January 20^th^ 2021 | February 15^th^ 2021 |
| KEGG | February 14^th^ 2021 | February 15^th^ 2021 |
| WikiPathways | January 20^th^ 2021 | February 15^th^ 2021 |
| PhosphoSitePlus (KS) | January 20^th^ 2021 | February 15^th^ 2021 |
| PhosphoSitePlus (full) | January 20^th^ 2021 | February 15^th^ 2021 |
| HPRD | January 20^th^ 2021 | February 15^th^ 2021 |
| IMEX | November 6th 2019 | February 15^th^ 2021 |
| GO category | January 20^th^ 2021 | February 15^th^ 2021 |
| UniProt category | January 20^th^ 2021 | February 15^th^ 2021 |
| UniProt | January 20^th^ 2021 | February 15^th^ 2021 |
| qPhos | January 13th 2020 | February 15^th^ 2021 |
