# Supplementary material for: Measuring pathway database coverage of the phosphoproteome
Source: PeerJ. 2021 May 25;9:e11298. doi: 10.7717/peerj.11298 (PMC8162239; doi:10.7717/peerj.11298)
Supplement: Supplemental Information 11 [file peerj-09-11298-s011.docx]

**Supplementary Table 3: Top Six QPhos Cell Lines with the Most PubMed ID’s.**

| **Cell Line name** | **Number of PubMed ID’s** | **Protein number** | **Phosphorylation number** |
| --- | --- | --- | --- |
| **Hela** | 15 | 9,578 | 67,759 |
| **HEK 293** | 8 | 6,826 | 31,597 |
| **Hela S3** | 6 | 5,870 | 29,755 |
| **Jurkat** | 5 | 5,316 | 27,499 |
| **MCF-7** | 8 | 5,000 | 25,606 |
| **MCF-10a** | 5 | 2,235 | 8,941 |
