# Supplementary material for: Measuring pathway database coverage of the phosphoproteome
Source: PeerJ. 2021 May 25;9:e11298. doi: 10.7717/peerj.11298 (PMC8162239; doi:10.7717/peerj.11298)
Supplement: Supplemental Information 12 [file peerj-09-11298-s012.docx]

**Supplementary Table 4: Experimental Overlap of all qPhos Conditions with Analysed Databases on the Proteomic level.**

| Intersection | HPRD | Bio-GRID | React-ome | KEGG | Wiki-pathways | Phospho-SitePlus | SIG-NOR |
| --- | --- | --- | --- | --- | --- | --- | --- |
| UID_184A1_NoIso.txt | 91 | 117 | 95 | 77 | 76 | 76 | 86 |
| UID_6-10B_NoIso.txt | 48 | 69 | 47 | 34 | 41 | 34 | 40 |
| UID_A-204_NoIso.txt | 1525 | 2551 | 1629 | 1111 | 1177 | 1001 | 1165 |
| UID_A-431_NoIso.txt | 2366 | 3987 | 2538 | 1733 | 1844 | 1423 | 1779 |
| UID_A-498_NoIso.txt | 1218 | 1905 | 1248 | 926 | 968 | 822 | 958 |
| UID_A-549_NoIso.txt | 1135 | 1820 | 1224 | 822 | 907 | 806 | 908 |
| UID_Akata(EBV+)_NoIso.txt | 1408 | 2277 | 1490 | 954 | 1066 | 885 | 1087 |
| UID_AML_NoIso.txt | 512 | 813 | 532 | 355 | 382 | 389 | 408 |
| UID_ARPE-19_NoIso.txt | 757 | 1187 | 764 | 573 | 617 | 556 | 628 |
| UID_BaF3_NoIso.txt | 545 | 793 | 626 | 519 | 502 | 358 | 437 |
| UID_blood_NoIso.txt | 1349 | 2179 | 1530 | 1153 | 1154 | 807 | 1074 |
| UID_BOEC_NoIso.txt | 1659 | 2742 | 1740 | 1215 | 1292 | 1055 | 1300 |
| UID_brain_NoIso.txt | 888 | 1462 | 990 | 741 | 752 | 490 | 686 |
| UID_breast_cancer_NoIso.txt | 3917 | 6938 | 4413 | 3099 | 3205 | 2014 | 2824 |
| UID_BT-474_NoIso.txt | 1693 | 2788 | 1776 | 1209 | 1291 | 1063 | 1301 |
| UID_CAL-27_NoIso.txt | 244 | 353 | 266 | 230 | 243 | 203 | 238 |
| UID_CCRF-CEM_NoIso.txt | 1037 | 1649 | 1115 | 746 | 821 | 740 | 823 |
| UID_CD4+T_cell_NoIso.txt | 1409 | 2303 | 1525 | 1062 | 1111 | 999 | 1148 |
| UID_CL1-0_NoIso.txt | 463 | 717 | 464 | 291 | 315 | 318 | 334 |
| UID_DFC_NoIso.txt | 1022 | 1653 | 1047 | 759 | 829 | 708 | 826 |
| UID_DG-75_NoIso.txt | 895 | 1436 | 956 | 674 | 729 | 612 | 706 |
| UID_DLD-1_NoIso.txt | 711 | 1070 | 711 | 500 | 562 | 531 | 571 |
| UID_Erythrocyte_NoIso.txt | 60 | 106 | 79 | 61 | 52 | 39 | 55 |
| UID_GB-2_NoIso.txt | 889 | 1369 | 914 | 647 | 707 | 592 | 716 |
| UID_GIST-T1_NoIso.txt | 95 | 154 | 112 | 96 | 91 | 88 | 89 |
| UID_Glioblastoma_initiating_cell_NoIso.txt | 781 | 1229 | 801 | 554 | 624 | 562 | 665 |
| UID_HaCaT_NoIso.txt | 2072 | 3400 | 2205 | 1488 | 1608 | 1258 | 1550 |
| UID_HAEC_NoIso.txt | 60 | 97 | 72 | 52 | 54 | 62 | 62 |
| UID_HCT_116_NoIso.txt | 2794 | 4736 | 3017 | 2072 | 2187 | 1592 | 2072 |
| UID_HEBC_NoIso.txt | 37 | 48 | 40 | 34 | 35 | 40 | 41 |
| UID_HEK293_NoIso.txt | 3551 | 6369 | 4040 | 2841 | 2836 | 1822 | 2562 |
| UID_HeLa_Kyoto_NoIso.txt | 900 | 1433 | 932 | 638 | 679 | 659 | 715 |
| UID_HeLa_NoIso.txt | 4672 | 8758 | 5551 | 3954 | 3882 | 2130 | 3268 |
| UID_HeLa_S3_NoIso.txt | 3178 | 5482 | 3615 | 2602 | 2526 | 1678 | 2258 |
| UID_Hep-G2_NoIso.txt | 1036 | 1618 | 1118 | 782 | 804 | 668 | 764 |
| UID_HES-3_NoIso.txt | 144 | 200 | 163 | 146 | 143 | 128 | 141 |
| UID_HFF_NoIso.txt | 1644 | 2750 | 1739 | 1238 | 1292 | 1009 | 1255 |
| UID_HL-60_NoIso.txt | 1745 | 2891 | 1901 | 1293 | 1352 | 1117 | 1352 |
| UID_HNSCC_NoIso.txt | 1315 | 2136 | 1373 | 926 | 1008 | 867 | 1010 |
| UID_hSAEC_NoIso.txt | 371 | 571 | 354 | 262 | 292 | 273 | 305 |
| UID_HSF_NoIso.txt | 55 | 101 | 57 | 44 | 50 | 53 | 57 |
| UID_HT-29_NoIso.txt | 171 | 265 | 172 | 118 | 145 | 143 | 149 |
| UID_HT22_NoIso.txt | 816 | 1290 | 891 | 671 | 711 | 626 | 749 |
| UID_hTERT-RPE1_NoIso.txt | 3190 | 5566 | 3521 | 2425 | 2515 | 1714 | 2297 |
| UID_HUES_9_NoIso.txt | 1977 | 3165 | 2128 | 1488 | 1520 | 1131 | 1418 |
| UID_HUVEC-C_NoIso.txt | 106 | 155 | 99 | 64 | 86 | 82 | 86 |
| UID_J_gamma1_NoIso.txt | 630 | 950 | 703 | 553 | 563 | 427 | 531 |
| UID_J_VAv1_NoIso.txt | 372 | 535 | 423 | 354 | 351 | 246 | 315 |
| UID_J14_NoIso.txt | 158 | 220 | 174 | 157 | 151 | 122 | 143 |
| UID_Jurkat_E6.1_NoIso.txt | 2029 | 3397 | 2195 | 1488 | 1592 | 1296 | 1540 |
| UID_Jurkat_NoIso.txt | 2968 | 5029 | 3231 | 2236 | 2334 | 1654 | 2138 |
| UID_Jurkat(HIV_expressing)_NoIso.txt | 1315 | 2163 | 1406 | 966 | 1024 | 885 | 1025 |
| UID_K-562_NoIso.txt | 923 | 1475 | 982 | 679 | 713 | 667 | 738 |
| UID_KG-1_NoIso.txt | 1580 | 2577 | 1707 | 1151 | 1210 | 1057 | 1233 |
| UID_Kit225_NoIso.txt | 911 | 1491 | 1009 | 709 | 768 | 655 | 788 |
| UID_liver_NoIso.txt | 2414 | 4092 | 2703 | 1882 | 1983 | 1406 | 1793 |
| UID_LM-MEL-28_NoIso.txt | 690 | 1058 | 686 | 465 | 504 | 498 | 533 |
| UID_LNCaP_clone_FGC_NoIso.txt | 390 | 612 | 403 | 271 | 311 | 306 | 326 |
| UID_lung_NoIso.txt | 418 | 658 | 423 | 306 | 347 | 306 | 344 |
| UID_Macrophage_NoIso.txt | 467 | 766 | 492 | 368 | 386 | 359 | 390 |
| UID_MCF-10A_NoIso.txt | 1297 | 2116 | 1367 | 946 | 1028 | 881 | 1027 |
| UID_MCF-7_NoIso.txt | 2726 | 4716 | 2953 | 1991 | 2099 | 1537 | 1987 |
| UID_MDA-MB-231_NoIso.txt | 1445 | 2379 | 1533 | 1029 | 1133 | 960 | 1140 |
| UID_Melanocytes_NoIso.txt | 592 | 937 | 603 | 427 | 458 | 442 | 480 |
| UID_MRC-5_NoIso.txt | 537 | 779 | 546 | 404 | 427 | 369 | 424 |
| UID_myocardial_NoIso.txt | 250 | 411 | 253 | 194 | 208 | 171 | 199 |
| UID_myometrial_NoIso.txt | 11 | 14 | 8 | 7 | 7 | 6 | 8 |
| UID_NB4_NoIso.txt | 808 | 1306 | 895 | 577 | 632 | 571 | 656 |
| UID_NCI-H1299_NoIso.txt | 1654 | 2767 | 1768 | 1185 | 1296 | 1079 | 1293 |
| UID_NCI-H2228_NoIso.txt | 2855 | 4959 | 3154 | 2182 | 2212 | 1615 | 2080 |
| UID_NCI-H292_NoIso.txt | 279 | 448 | 297 | 226 | 225 | 228 | 248 |
| UID_NCI-H358_NoIso.txt | 579 | 889 | 584 | 426 | 468 | 446 | 478 |
| UID_NCI-H929_NoIso.txt | 1675 | 2740 | 1805 | 1270 | 1335 | 1071 | 1293 |
| UID_nfHCC_NoIso.txt | 22 | 31 | 22 | 15 | 23 | 21 | 20 |
| UID_NHA_NoIso.txt | 1482 | 2412 | 1561 | 1086 | 1190 | 977 | 1176 |
| UID_NHDF_NoIso.txt | 115 | 157 | 103 | 77 | 87 | 79 | 88 |
| UID_NSCLC_NoIso.txt | 904 | 1428 | 942 | 633 | 740 | 610 | 750 |
| UID_pancreas_NoIso.txt | 1189 | 2031 | 1316 | 947 | 1018 | 804 | 1004 |
| UID_PaSC_NoIso.txt | 2158 | 3609 | 2290 | 1539 | 1677 | 1330 | 1655 |
| UID_PC-9_NoIso.txt | 113 | 164 | 126 | 94 | 101 | 78 | 101 |
| UID_placenta_NoIso.txt | 740 | 1379 | 892 | 643 | 650 | 422 | 594 |
| UID_SCC-9_NoIso.txt | 1973 | 3241 | 2099 | 1429 | 1521 | 1234 | 1508 |
| UID_SGC-7901_NoIso.txt | 158 | 309 | 193 | 141 | 138 | 84 | 115 |
| UID_SH-SY5Y_NoIso.txt | 967 | 1584 | 1035 | 710 | 783 | 700 | 804 |
| UID_Shef4_NoIso.txt | 287 | 446 | 294 | 199 | 231 | 244 | 254 |
| UID_SK-BR-3_NoIso.txt | 135 | 209 | 142 | 105 | 118 | 121 | 129 |
| UID_Skeletal_muscle_NoIso.txt | 60 | 81 | 60 | 42 | 50 | 43 | 47 |
| UID_skin_NoIso.txt | 159 | 225 | 174 | 120 | 142 | 131 | 149 |
| UID_SW1736_NoIso.txt | 535 | 810 | 540 | 365 | 425 | 421 | 432 |
| UID_SW48_NoIso.txt | 139 | 206 | 138 | 104 | 117 | 117 | 116 |
| UID_SW480_NoIso.txt | 34 | 43 | 31 | 22 | 31 | 35 | 31 |
| UID_THP-1_NoIso.txt | 638 | 1000 | 661 | 463 | 498 | 465 | 520 |
| UID_TIG-3_NoIso.txt | 2196 | 3647 | 2342 | 1631 | 1741 | 1324 | 1689 |
| UID_U-251MG_NoIso.txt | 463 | 664 | 449 | 323 | 379 | 361 | 371 |
| UID_U-937_NoIso.txt | 844 | 1407 | 919 | 631 | 693 | 585 | 698 |
| UID_U266B1_NoIso.txt | 72 | 117 | 83 | 69 | 60 | 55 | 60 |
| UID_U2OS_NoIso.txt | 2358 | 3925 | 2507 | 1734 | 1873 | 1389 | 1753 |
| UID_venous_blood_NoIso.txt | 256 | 397 | 288 | 218 | 241 | 203 | 238 |
| UID_WM239A_NoIso.txt | 2792 | 4781 | 3007 | 2062 | 2202 | 1553 | 2051 |
