# Supplementary material for: Measuring pathway database coverage of the phosphoproteome
Source: PeerJ. 2021 May 25;9:e11298. doi: 10.7717/peerj.11298 (PMC8162239; doi:10.7717/peerj.11298)
Supplement: Supplemental Information 13 [file peerj-09-11298-s013.docx]

**Supplementary Table 5: Experimental Overlap of all qPhos Conditions with Analysed Databases on the Phosphoproteomic Level.**

| Intersection | HPRD | BioGRID | PhosphoSitePlus | Reactome | SIGNOR |
| --- | --- | --- | --- | --- | --- |
| UID_MOD_184A1_NoIso.txt | 120 | 12 | 36 | 61 | 65 |
| UID_MOD_6-10B_NoIso.txt | 80 | 29 | 0 | 16 | 14 |
| UID_MOD_A-204_NoIso.txt | 3843 | 1309 | 105 | 767 | 465 |
| UID_MOD_A-431_NoIso.txt | 6387 | 1882 | 180 | 1202 | 734 |
| UID_MOD_A-498_NoIso.txt | 3313 | 835 | 137 | 631 | 458 |
| UID_MOD_A-549_NoIso.txt | 2886 | 899 | 112 | 578 | 390 |
| UID_MOD_Akata(EBV+)_NoIso.txt | 2979 | 877 | 66 | 611 | 348 |
| UID_MOD_AML_NoIso.txt | 980 | 361 | 30 | 229 | 152 |
| UID_MOD_ARPE-19_NoIso.txt | 1290 | 520 | 70 | 331 | 250 |
| UID_MOD_BaF3_NoIso.txt | 230 | 12 | 34 | 87 | 68 |
| UID_MOD_blood_NoIso.txt | 1987 | 634 | 106 | 534 | 364 |
| UID_MOD_BOEC_NoIso.txt | 4353 | 1437 | 143 | 891 | 576 |
| UID_MOD_brain_NoIso.txt | 1414 | 339 | 49 | 298 | 236 |
| UID_MOD_breast_cancer_NoIso.txt | 10698 | 2660 | 340 | 2058 | 1263 |
| UID_MOD_BT-474_NoIso.txt | 4049 | 1269 | 143 | 855 | 542 |
| UID_MOD_CAL-27_NoIso.txt | 357 | 28 | 69 | 148 | 148 |
| UID_MOD_CCRF-CEM_NoIso.txt | 1857 | 637 | 81 | 481 | 291 |
| UID_MOD_CD4+T_cell_NoIso.txt | 2767 | 819 | 89 | 631 | 405 |
| UID_MOD_CL1-0_NoIso.txt | 1241 | 374 | 27 | 222 | 165 |
| UID_MOD_DFC_NoIso.txt | 1687 | 657 | 77 | 410 | 290 |
| UID_MOD_DG-75_NoIso.txt | 1553 | 454 | 60 | 313 | 212 |
| UID_MOD_DLD-1_NoIso.txt | 1394 | 453 | 91 | 388 | 290 |
| UID_MOD_Erythrocyte_NoIso.txt | 92 | 35 | 1 | 20 | 9 |
| UID_MOD_GB-2_NoIso.txt | 1450 | 572 | 75 | 415 | 298 |
| UID_MOD_GIST-T1_NoIso.txt | 88 | 9 | 25 | 35 | 34 |
| UID_MOD_Glioblastoma_initiating_cell_NoIso.txt | 1367 | 407 | 58 | 325 | 250 |
| UID_MOD_HaCaT_NoIso.txt | 5219 | 1347 | 139 | 968 | 584 |
| UID_MOD_HAEC_NoIso.txt | 78 | 37 | 14 | 37 | 31 |
| UID_MOD_HCT_116_NoIso.txt | 6831 | 1562 | 255 | 1344 | 839 |
| UID_MOD_HEBC_NoIso.txt | 39 | 7 | 14 | 25 | 26 |
| UID_MOD_HEK293_NoIso.txt | 9659 | 2366 | 273 | 1663 | 1023 |
| UID_MOD_HeLa_Kyoto_NoIso.txt | 2006 | 654 | 65 | 455 | 289 |
| UID_MOD_HeLa_NoIso.txt | 16645 | 2819 | 354 | 2626 | 1509 |
| UID_MOD_HeLa_S3_NoIso.txt | 14442 | 1970 | 243 | 1699 | 995 |
| UID_MOD_Hep-G2_NoIso.txt | 1833 | 627 | 45 | 340 | 231 |
| UID_MOD_HES-3_NoIso.txt | 181 | 17 | 59 | 93 | 93 |
| UID_MOD_HFF_NoIso.txt | 3420 | 1031 | 108 | 637 | 407 |
| UID_MOD_HL-60_NoIso.txt | 3905 | 1152 | 131 | 839 | 515 |
| UID_MOD_HNSCC_NoIso.txt | 2025 | 821 | 88 | 499 | 316 |
| UID_MOD_hSAEC_NoIso.txt | 515 | 226 | 34 | 159 | 110 |
| UID_MOD_HSF_NoIso.txt | 40 | 13 | 8 | 37 | 22 |
| UID_MOD_HT-29_NoIso.txt | 241 | 109 | 15 | 83 | 60 |
| UID_MOD_HT22_NoIso.txt | 1278 | 396 | 90 | 369 | 270 |
| UID_MOD_hTERT-RPE1_NoIso.txt | 11021 | 2312 | 269 | 1925 | 1098 |
| UID_MOD_HUES_9_NoIso.txt | 6118 | 1417 | 122 | 937 | 569 |
| UID_MOD_HUVEC-C_NoIso.txt | 92 | 25 | 4 | 28 | 17 |
| UID_MOD_J_gamma1_NoIso.txt | 434 | 27 | 56 | 156 | 123 |
| UID_MOD_J_VAv1_NoIso.txt | 265 | 16 | 41 | 97 | 85 |
| UID_MOD_J14_NoIso.txt | 142 | 14 | 25 | 53 | 50 |
| UID_MOD_Jurkat_E6.1_NoIso.txt | 7058 | 1691 | 197 | 1219 | 757 |
| UID_MOD_Jurkat_NoIso.txt | 9496 | 2082 | 320 | 1827 | 1071 |
| UID_MOD_Jurkat(HIV_expressing)_NoIso.txt | 2491 | 777 | 68 | 489 | 321 |
| UID_MOD_K-562_NoIso.txt | 2228 | 738 | 61 | 436 | 260 |
| UID_MOD_KG-1_NoIso.txt | 5091 | 1278 | 138 | 917 | 533 |
| UID_MOD_Kit225_NoIso.txt | 1408 | 344 | 66 | 332 | 237 |
| UID_MOD_liver_NoIso.txt | 6601 | 1723 | 183 | 1089 | 696 |
| UID_MOD_LM-MEL-28_NoIso.txt | 1560 | 494 | 52 | 339 | 236 |
| UID_MOD_LNCaP_clone_FGC_NoIso.txt | 825 | 293 | 37 | 211 | 156 |
| UID_MOD_lung_NoIso.txt | 822 | 229 | 19 | 178 | 124 |
| UID_MOD_Macrophage_NoIso.txt | 614 | 278 | 27 | 174 | 108 |
| UID_MOD_MCF-10A_NoIso.txt | 3797 | 1068 | 166 | 757 | 525 |
| UID_MOD_MCF-7_NoIso.txt | 9404 | 2398 | 248 | 1578 | 1005 |
| UID_MOD_MDA-MB-231_NoIso.txt | 3240 | 1056 | 86 | 654 | 400 |
| UID_MOD_Melanocytes_NoIso.txt | 1424 | 469 | 45 | 282 | 199 |
| UID_MOD_MRC-5_NoIso.txt | 1637 | 349 | 45 | 282 | 181 |
| UID_MOD_myocardial_NoIso.txt | 431 | 105 | 20 | 123 | 101 |
| UID_MOD_myometrial_NoIso.txt | 14 | 1 | 2 | 5 | 2 |
| UID_MOD_NB4_NoIso.txt | 1634 | 551 | 37 | 309 | 190 |
| UID_MOD_NCI-H1299_NoIso.txt | 3686 | 1013 | 79 | 687 | 386 |
| UID_MOD_NCI-H2228_NoIso.txt | 7946 | 2251 | 234 | 1611 | 939 |
| UID_MOD_NCI-H292_NoIso.txt | 275 | 106 | 20 | 82 | 69 |
| UID_MOD_NCI-H358_NoIso.txt | 757 | 299 | 41 | 221 | 154 |
| UID_MOD_NCI-H929_NoIso.txt | 3827 | 1228 | 139 | 854 | 556 |
| UID_MOD_nfHCC_NoIso.txt | 35 | 12 | 1 | 10 | 8 |
| UID_MOD_NHA_NoIso.txt | 2713 | 1033 | 103 | 690 | 415 |
| UID_MOD_NHDF_NoIso.txt | 137 | 52 | 4 | 27 | 20 |
| UID_MOD_NSCLC_NoIso.txt | 964 | 361 | 22 | 200 | 118 |
| UID_MOD_pancreas_NoIso.txt | 2455 | 825 | 73 | 525 | 343 |
| UID_MOD_PaSC_NoIso.txt | 5979 | 1448 | 141 | 1012 | 618 |
| UID_MOD_PC-9_NoIso.txt | 116 | 9 | 25 | 53 | 48 |
| UID_MOD_placenta_NoIso.txt | 584 | 141 | 19 | 145 | 116 |
| UID_MOD_Sample_NoIso.txt | 0 | 0 | 0 | 0 | 0 |
| UID_MOD_SCC-9_NoIso.txt | 6259 | 1663 | 174 | 1114 | 700 |
| UID_MOD_SGC-7901_NoIso.txt | 81 | 29 | 9 | 21 | 20 |
| UID_MOD_SH-SY5Y_NoIso.txt | 1487 | 531 | 80 | 403 | 270 |
| UID_MOD_Shef4_NoIso.txt | 387 | 120 | 32 | 128 | 85 |
| UID_MOD_SK-BR-3_NoIso.txt | 247 | 72 | 19 | 84 | 66 |
| UID_MOD_Skeletal_muscle_NoIso.txt | 116 | 41 | 2 | 29 | 32 |
| UID_MOD_skin_NoIso.txt | 216 | 77 | 21 | 89 | 72 |
| UID_MOD_SW1736_NoIso.txt | 1089 | 373 | 54 | 287 | 198 |
| UID_MOD_SW48_NoIso.txt | 200 | 64 | 11 | 43 | 24 |
| UID_MOD_SW480_NoIso.txt | 39 | 18 | 6 | 18 | 13 |
| UID_MOD_THP-1_NoIso.txt | 1625 | 553 | 56 | 329 | 226 |
| UID_MOD_TIG-3_NoIso.txt | 5451 | 1484 | 182 | 1075 | 652 |
| UID_MOD_U-251MG_NoIso.txt | 757 | 271 | 34 | 223 | 155 |
| UID_MOD_U-937_NoIso.txt | 1170 | 391 | 46 | 279 | 192 |
| UID_MOD_U266B1_NoIso.txt | 71 | 24 | 0 | 21 | 15 |
| UID_MOD_U2OS_NoIso.txt | 5574 | 1609 | 191 | 1193 | 713 |
| UID_MOD_venous_blood_NoIso.txt | 374 | 112 | 43 | 131 | 113 |
| UID_MOD_WM239A_NoIso.txt | 7329 | 1849 | 206 | 1329 | 811 |
